# Profiling Cisplatin Resistance in Head and Neck Cancer: A Critical Role of the VRAC Ion Channel for Chemoresistance

**DOI:** 10.3390/cancers13194831

**Published:** 2021-09-27

**Authors:** Svenja Siemer, Torsten Fauth, Paul Scholz, Yara Al-Zamel, Aya Khamis, Désirée Gül, Laura Freudelsperger, Barbara Wollenberg, Sven Becker, Roland H. Stauber, Jan Hagemann

**Affiliations:** 1 Department of Otorhinolaryngology Head and Neck Surgery, Molecular and Cellular Oncology, University Medical Center, 55131 Mainz, Germany; Svenja.siemer@uni-mainz.de (S.S.); yalzamel@students.uni-mainz.de (Y.A.-Z.); ayakhamis@uni-mainz.de (A.K.); Desiree.guel@unimedizin-mainz.de (D.G.); laura.freudelsperger@unimedizin-mainz.de (L.F.); sven.becker@med.uni-tuebingen.de (S.B.); jan.hagemann@unimedizin-mainz.de (J.H.); 2 BRAIN Biotech AG, 64673 Zwingenberg, Germany; tf@brain-biotech.de (T.F.); ps@brain-biotech.de (P.S.); 3 Department of Otorhinolaryngology Head and Neck Surgery, University Hospital Klinikum Rechts der Isar, 81675 Munich, Germany; barbara.wollenberg@tum.de; 4 Department of Otorhinolaryngology, University Medical Center Tuebingen, 72076 Tuebingen, Germany

**Keywords:** chemotherapy resistance, HNSCC, tumor therapy, drug transporter, personalized medicine

## Abstract

**Simple Summary:**

Treatment success of head and neck cancers (HNSCC) is often hindered by chemoresistance. In this study, next-generation sequencing transcriptomics and CRISPR/Cas9 knockout strategies were used to identify cisplatin resistance mechanisms and potential (personalized) biomarkers. Moreover, employing a tiered experimental pipeline, the cisplatin uptake transporter VRAC was found to be critical for cisplatin sensitivity and specificity in 2D/3D-HNSCC cell culture models as well as for tumor relapses in the clinical setting. Our study suggests exploiting VRAC as a potential drug target as well as a personalized prognostic biomarker to improve the treatment of HNSCC patients in the future.

**Abstract:**

Treatment success of head and neck cancers (HNSCC) is often hindered by tumor relapses due to therapy resistances. This study aimed at profiling cisplatin resistance mechanisms and identifying biomarkers potentially suitable as drug targets and for patient stratification. Bioinformatic analyses of suggested resistance factors in a cohort of 565 HNSCC patients identified the VRAC ion channel as a clinically relevant indicator for recurrent diseases following radiochemotherapy (*p* = 0.042). Other drug import/export transporters, such as CTR1, OCT1, or MRP1, were found to be less relevant. To experimentally verify VRAC’s critical role for cisplatin resistance, we used CRISPR/Cas9 knockout resulting in cisplatin-resistant HNSCC cells, which could be resensitized by VRAC expression. Next-generation sequencing further underlined VRAC’s importance and identified VRAC-regulated signaling networks, potentially also contributing to cisplatin resistance. CTR1, OCT1, or MRP1 did not contribute to increased cisplatin resistance. In addition to two-dimensional HNSCC models, three-dimensional tumor spheroid cultures confirmed VRAC’s unique role for cisplatin sensitivity. Here, resistance correlated with DNA damage and downstream apoptosis. The cisplatin specificity of the identified VRAC pathway was verified by testing paclitaxel and doxorubicin. Our results were independently confirmed in naturally occurring, cisplatin-resistant HNSCC cancer cell models. Collectively, we here demonstrate VRAC’s role for cisplatin resistance in HNSCC and its relevance as a potential drug target and/or prognostic biomarker for chemotherapy resistance.

## 1. Introduction

Cancers of the head and neck range among the ten most frequent malignant diseases, and head and neck squamous cell carcinomas (HNSCCs) account for roughly 90% among them. Despite extensive and radical primary treatment options, the median 5-year overall survival averages around 65% with a range of 30–85%, depending on tumor stage. HNSCC treatment options with intention to cure mainly include either surgical removal with or without adjuvant (chemo)radiation or radiotherapy with concurrent chemotherapy. There is increasing evidence indicating that the expression of molecular markers, primarily p16 as a surrogate for human papilloma virus (HPV)-associated carcinogenesis, could be decisive when choosing the right first-line treatment regimen [1]. However, highly heterogeneous cancer cell populations [2] in HNSCC did prevent extensive efforts to establish other predictive markers on the road to personalized therapy.

**Figure 1:**
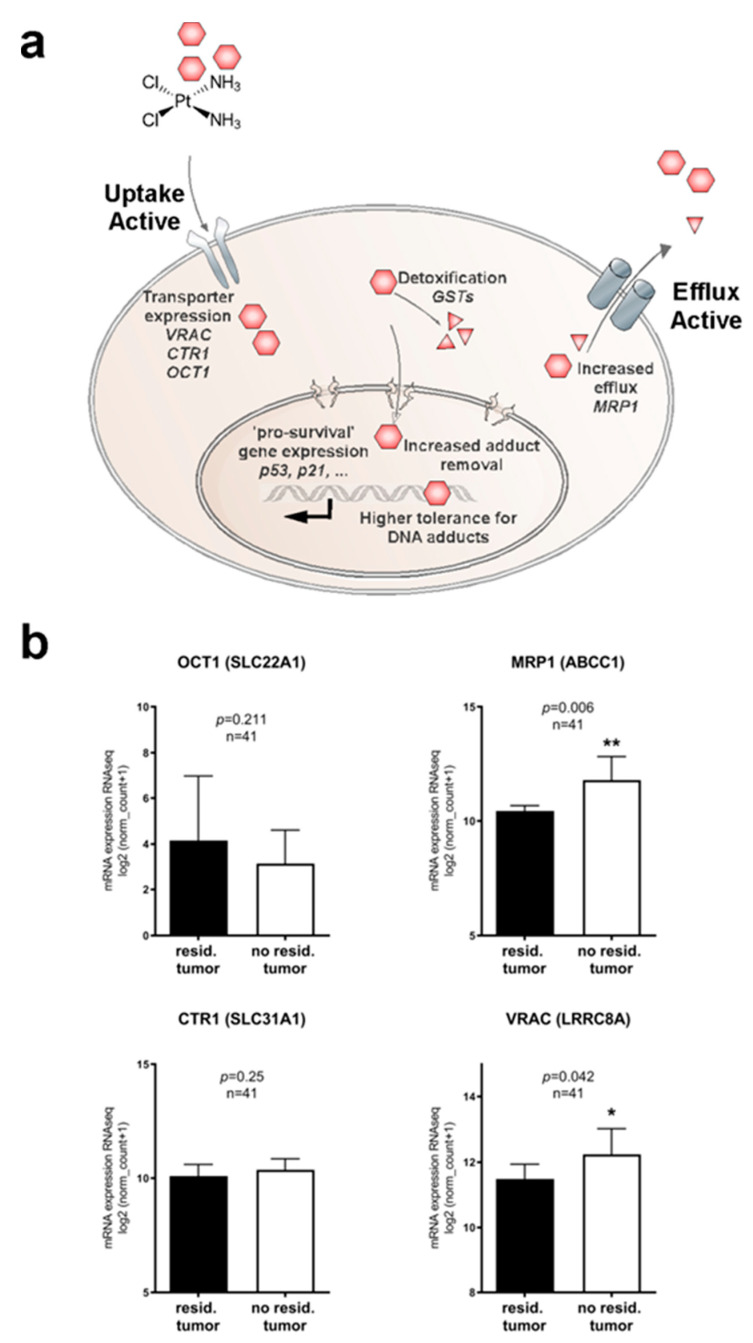
Molecular pathways and potential clinically relevant players contributing to cisplatin resistance in HNSCC. (**a**) Cartoon depicting mechanisms potentially involved in cisplatin resistance. Reduced intracellular drug concentrations can be a consequence of reduced uptake, accelerated efflux, or intracellular detoxification. Additionally, improved DNA repair and various (indirect) pro-survival pathways may improve cancer cells’ ability to cope with cisplatin toxicity. (**b**) Bioinformatic identification of potential clinically relevant drug transporters in the transcriptomics dataset of HNSCC patients from *The Cancer Genome Atlas* (TCGA) (*n* = 565). Correlation of drug transporter transcription levels with residual tumors after first-line chemoradiotherapy and full clinical documentation (*n* = 41) were assessed. Low expression of drug import channels, i.e., reduced uptake of cisplatin, is expected to favor cancer cell survival and thus, tumor recurrences. We found an unexpected trend of decreased expression of OCT1, and expression levels of the drug uptake transporter CTR1 remain similar. However, enhanced expression of the drug export transporter MRP1 and increased expression of the drug uptake transporter VRAC significantly correlated with lower tumor recurrences. *, *p* < 0.05; **, *p* < 0.01.

First-line chemotherapy of HNSCCs is predominantly platinum-based with cisplatin being the primary option. While recently, the field could see promising results by using α-PD-1 checkpoint inhibitor pembrolizumab [3] as platinum alternative in a subset of cases, platinum remains the most effective and economic therapy for the majority of HNSCC patients. On the other hand, therapy resistance and associated subsequent near or distant relapses are still common and are associated with high patient morbidity and a median survival of only 10 months [4,5]. The development of resistance mechanisms toward (platinum-based) chemotherapeutics is clearly a complex multilevel process, potentially including alterations in drug import and export, apoptosis initiation/inhibition, improved drug detoxification, as well as DNA repair [6,7,8,9,10] (Figure 1a). The impact of pre-existing resistance factors due to high mutational loads of HNSCCs compared to dynamic adaption on transcriptome level remains unclear. Thus, resistance to chemotherapeutics is manifold, complex, and not yet fully understood. Previous studies aimed to identify gene signatures for HNSCC, allowing predicting patients’ therapy responses, pathobiology, and survival. However, the suggested signatures still failed to be used in the clinical routine to guide (targeted therapy) treatments for all patients [11,12]. Alongside methodological complications, the fact that HNSCC is characterized by highly heterogeneous tumors and anatomical localizations makes the selection of relevant patient subpopulations difficult and prevents rapid progress. Investigating crucial cellular components involved in the transport of platinum-based drugs could lead to further understanding of platinum resistance here. Transporters facilitating active influx and efflux of platinum drugs seem to be especially promising candidates in this context, as they have a direct impact on final intracellular drug concentrations (Figure 1a). A variety of drug transporters such as CTR1, OCT1, VRAC, and MRP1 were suggested to be involved in the transport and resistance of platinum-based drugs, with passive trans-membrane diffusion playing only a subordinate role. Particularly, the pioneering work by the Jentsch group not only demonstrated VRACS’s relevance for cisplatin resistance but also showed that the subunit composition of VRAC plays additional roles for drug uptake properties [13,14,15,16,17,18,19]. Taking into consideration these complexities, we argued that it is necessary to employ a tiered experimental pipeline from in silico to analytical and in vitro to understand and potentially overcome cisplatin resistance in HNSCC.

In our study, we first used TCGA data analyses of genomic and transcriptomic alterations in drug transport channels to correlate their relevance with clinical phenotypes. Subsequently, we experimentally identified the ion channel VRAC as a critical component for cisplatin-specific uptake and resistance and confirmed its clinical relevance. Our results strongly suggest exploiting VRAC as a potential drug target as well as a personalized prognostic biomarker to improve the treatment of HNSCC patients in the future.

## 2. Results and Discussion

### 2.1. Identifying Clinically Relevant Players of Cisplatin Resistance

Cellular uptake and efflux processes of chemotherapeutics are certainly decisive for intracellular drug concentrations and ultimately cancer treatment success or failure (Figure 1a). Therefore, we analyzed the expression levels of transport channels suggested to be involved in cisplatin transport in the publicly available TCGA HNSCC dataset, comprising 565 cancer patients of various disease states and clinical backgrounds. Gene expression data of such candidates, e.g., CTR1 (gene *SLC31A1*), VRAC (gene *LRRC8A*), OCT1 (gene *SLC22A1*), or MRP1 (gene *ABCC1*) were extracted from the database and cut-offs for high and low expression were generated. To identify cisplatin therapy-specific relevance, we first focused on patients that underwent cisplatin-based chemoradiation as a first-line therapy. Limited by the availability of exact information regarding clinical treatments and remission status information, we were able to identify 41 cases. While there were no significant differences in residual tumor cases depending on expression level for transporters CTR1 and OCT1, low expression of the drug import channel VRAC and increased expression of the drug export transporter MRP1 significantly correlated with residual disease after chemoradiation in curative intent (*p* = 0.042 and *p* = 0.06 respectively; Figure 1b). However, the correlation of high expression of the drug efflux transporter MRP1 with improved chemoradiation is mechanistically hard to understand. In contrast, the observed correlation of cisplatin therapy success with high VRAC/LRRC8A expression, lowering intracellular cisplatin concentrations and thus promoting cancer cell survival, seems more relevant and suitable for further experimental investigations. Notably, when we analyzed overall survival for all patients irrespective of treatments, these trends were lost, showing various correlations of expression levels with disease prognosis (Figure S1). As VRAC is suspected to additionally support tumor cell survival pathways next to drug uptake, such heterogeneity can be expected in the absence of cisplatin selection pressure.

### 2.2. Profiling Cisplatin Sensitivity Pathways and Relevance of VRAC as a Critical Determinant for Cisplatin Resistance

**Figure 2:**
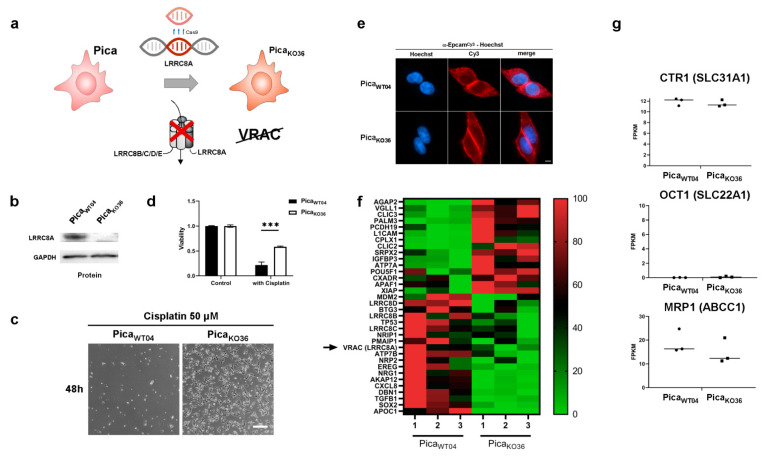
Profiling cisplatin sensitivity pathways underlines the relevance of VRAC as critical for cisplatin resistance. (**a**) Illustration of CRISPR/Cas9 technology to establish the VRAC-deficient, cisplatin-resistant, knockout cell line (Pica_KO36_). Scheme of the VRAC channel, consisting of six heteromeric subunits, indicated (**b**) Western blot to confirm the absence of LRRC8A protein expression in Pica_KO36_ cells. GAPDH served as loading control. (**c**) In contrast to WT cells, the VRAC-deficient cell line Pica_KO36_ is able to survive treatment with high cisplatin concentrations. Cells were treated with cisplatin (50 µM) for 48 h and imaged by live cell microscopy. Scale bar, 200 µm. (**d**) In contrast to WT cells, the VRAC-deficient cell line, Pica_KO36_, is significantly cisplatin resistant. Cells were exposed to cisplatin (20 µM) for 48 h and viability was normalized to untreated controls. ***, *p <* 0.005 (**e**) Pica_WT04_ and Pica_KO36_ cells show similar morphology. Fluorescence microscopy visualized EpCAM expression (stained with specific antibodies (red)), nuclei were stained with Hoechst (blue). Scale bar, 5 µm. (**f**) Applying RNASeq transcriptomics to identify cisplatin resistance players. Heatmap visualizing expression levels of potential cisplatin resistance-associated genes, which are differentially expressed in KO (Pica_KO36_) vs. cisplatin sensitive WT (Pica_WT04_) cells (green: downregulated, red: upregulated; full list of differentially expressed genes in Table S4). Constituting subunit of VRAC (LRRC8A) indicated. (**g**) Expression levels of previously described cisplatin-resistance channel proteins (CTR1, OCT1, MRP1; see Figure 1b) are unaffected in the HNSCC knockout cell line and thus are less relevant for HNSCC. RNA intensities as FPKM values are displayed. For uncropped blots, refer to Supplementary Materials.

Clearly, bioinformatic results are helpful for hypothesis building but need to be carefully confirmed experimentally employing HNSCC models and molecular mechanistic approaches. Thus, to further investigate VRAC’s unique role for cisplatin response on the cellular level and to test our hypothesis of a direct correlation of therapy response with VRAC expression, we next established an in vitro cell culture model. Here, CRISPR/Cas9 was used to specifically generate a complete LRRC8A knockout (KO) in HNSCC Pica cells, without inducing additional genetic alterations. LRRC8A is the only constituting subunit of the heteromeric transporter VRAC, leaving LRRC8A-KO cells unable to build functional VRAC channels [15,20] (Figure 2a). The Pica cell line was generated from a laryngeal squamous cell carcinoma with a novel protocol developed to mimic the tumor more precisely [21]. For maximal comparability and genetic homogeneity, different single cell clones were then generated from the LRRC8A-KO pool (Pica_KO36_) as well as from the wild-type (WT) cell line (Pica_WT04_). Genetic analysis on gDNA as well as cDNA level as well as Western blot analysis confirmed successful homozygous gene KO and absence of LRRC8A protein expression in cell line Pica_KO36_ (cells did not show an apparent difference to the initial cell population (Figure 2b,e). Probing cisplatin response of the cell lines, Pica_KO36_ cells could be shown to be significantly more resistant than WT cells, thus confirming a direct role of VRAC expression for cisplatin Figure 2c–d, Figure S3). Additionally, sequencing verified successful silencing of the respective genetic locus due to an insertion of a frameshift after amino acid 10, resulting in an additional stop codon after amino acid 66 of the LRRC8A protein (Figure S4, Tables S2 and S3). Macroscopically, the generated cells did not show an apparent difference to the initial cell population (Figure 2c). Probing cisplatin response of the cell lines, Pica_KO36_ cells could be shown to be significantly more resistant than WT cells, thus confirming a direct role of VRAC expression for cisplatin response (Figure 2d).

In order to further identify additional potential resistance networks, we also performed next-generation RNA sequencing transcriptomics on three samples per cell line. Subsequent bioinformatic analysis of differentially expressed genes revealed a list of candidates with potential impact on cisplatin resistance (Figure 2f, for raw data of significantly differentially expressed genes, see Table S4). Among these factors *TP53*, coding for the tumor suppressor protein p53 was downregulated in the knockout cells. Up to 80% of all cancers show aberrations of p53 expression, making it one of the most decisive proteins for cancer development. However, bearing in mind its multiple functions for DNA damage repair and correlation with tumor disease prognosis across several indications [22,23,24], this observation suggests a rather indirect mediator role of *TP53*. Likewise, we found differences in SOX2 expression levels. SOX2 has been reported also as an oncogenic transcription factor that is involved in the development of squamous cell carcinomas, and its increased expression was reported to correlate with drug resistance against tamoxifen in breast cancers [25,26,27]. Critically, changes in transcription factor expression as exemplified by these two candidates are able to further impact different downstream pathways and networks potentially resulting in vast changes of the cellular machinery. In contrast, growth factor receptors, e.g., EGFR, survival proteins such as survivin [28,29] or protease networks, e.g., the HNSCC-relevant protease taspase1 [30,31], were not affected (see Table S4). Importantly, other potential cisplatin transport channel candidates such as CTR1, OCT1, or MRP1 were neither up- or downregulated (Figure 2g), further underlining VRAC’s relevance for drug resistance. 

### 2.3. VRAC Expression in HNSCC Cancer Cells Is Key for Platinum Drug Sensitivity and Specificity

**Figure 3:**
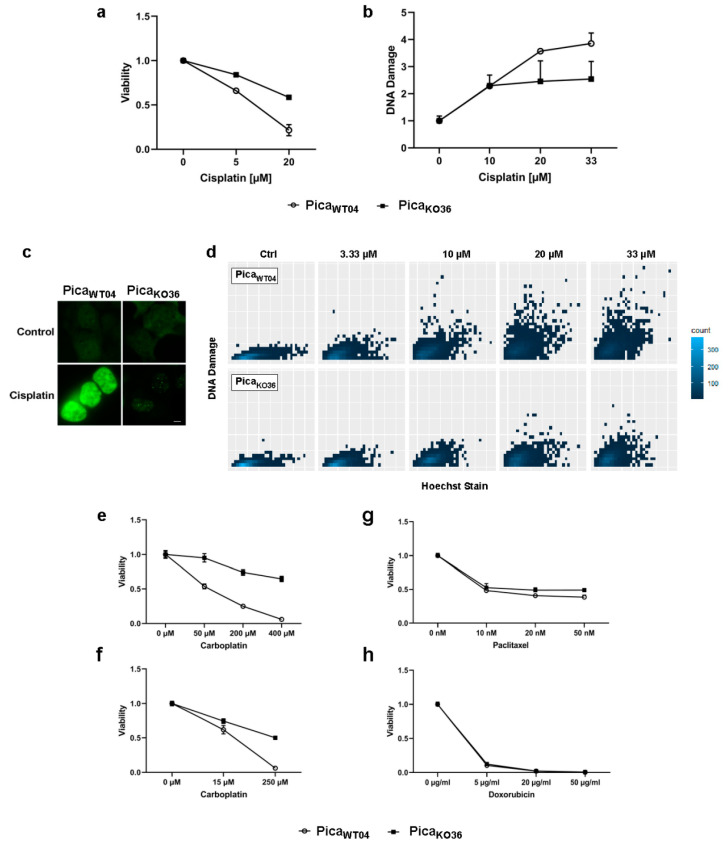
VRAC expression in HNSCC cancer cells is critical for platinum drug sensitivity and specificity. (**a**) VRAC-deficient Pica_KO36_ cells are dose-dependent resistant against cisplatin. Cells were treated for 48 h, and viability was normalized to untreated controls. (**b**–**d**) Resistant VRAC-deficient cells show lower number of cisplatin-induced DNA damage events (assayed as γH_2_AX damage foci) per cell. Cells were treated for 24 h and γH_2_AX foci detected by specific antibodies. (**b**) DNA damage automatically quantified by high-throughput microscopy and normalized to untreated controls. (**c**) Fluorescence microscopy to visualize DNA damage. DNA damage (γH_2_AX foci) stained by specific fluorescent antibodies (green). Scale bar, 5 µm. (**d**) Single-cell γH_2_AX foci were quantified via automatic high content screening microscope Array Scan VTI and plotted via ggplot2/R [32]. (**e**,**f**), VRAC-deficient Pica_KO36_ 2D cells (**e**) and 3D-tumor spheroids (**f**) are resistant against carboplatin treatment. Cells/spheroids were treated for 48/72 h and viability was normalized to untreated controls. (**g**,**h**) VRAC deficiency mediates cisplatin resistance but does not affect response to other cell-damaging drugs, such as paclitaxel or doxorubicin. Cells were treated for 48 h and viability was normalized to untreated controls.

As a strong candidate for cisplatin uptake and resistance identified in our dataset, we further analyzed the consequence of LRRC8A-KO on cisplatin toxicity. Cells were treated and revealed a significantly increased resistance toward cisplatin beyond clinically applied concentrations (<20 µM vs. ≈2 µM) (Figure 3a). Excluding methodical artefacts, the amount of induced DNA double-strand breaks per cell were additionally quantified as a second readout directly correlated to intracellular cisplatin levels. To this means, a protocol enabling the automated quantification of fluorescence signals in microscopy images of single cells immunofluorescently stained for DNA damage marker γH2AX was established using high content screening microscope Array Scan VTI. As shown in Figure 3, the KO cells showed significantly less double-strand breaks when compared to the respective WT cells both on the single-cell level (Figure 3d) as well as overall (Figure 3b), excluding methodical artefacts and confirming cellular resistance. Fluorescent microscopy confirmed the absence of background staining (Figure 3c). Notably, the transfection of LRRC8A expression plasmid reconstituted VRAC channel function and significantly resensitized the resistant LRRC8A-knockout Pica_KO36_ cells to cisplatin-mediated cell death (see Figure S5). These data provide independent strong evidence that mainly VRAC function is key for mediating cisplatin sensitivity.

Additionally, the absence of VRAC channel expression could be shown to confer resistance against carboplatin, which is an alternative platinum-based chemotherapeutic to cell line Pica_KO36_ (Figure 3e,f). In contrast, no cross-resistance was detectable for alternative chemotherapeutics doxorubicin and paclitaxel (Figure 3g,h), demonstrating a cisplatin-specific relevance of the identified VRAC-dependent molecular mechanism.

**Figure 4:**
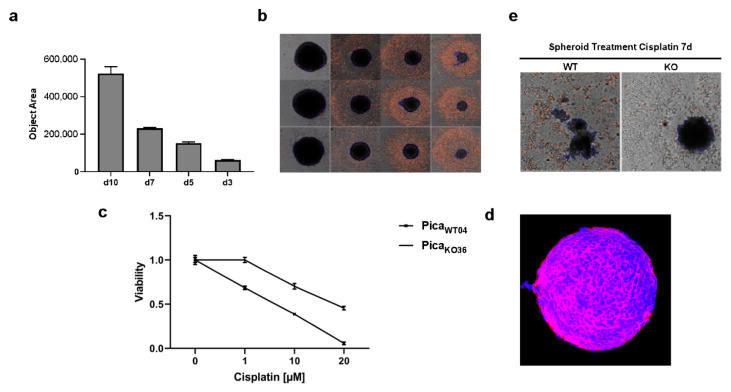
Absence of VRAC expression protects 3D tumor spheroids against cisplatin-induced cancer cell death. (**a**,**b**) Automated high content microscopy to visualize tumor spheroid growth. (**a**) Mean object sizes of spheroids (*n* = 8) automatically determined by high-content screening microscope Array Scan VTI. (**b**) Exemplary images of automatically detected regions of interest (ROI). Magnification, 5-fold. (**c**) Application of spheroids to confirm VRAC’s relevance for cisplatin resistance even in tumor mimicking 3D cultures. Spheroids were treated for 72 h, and viability was normalized to untreated controls. (**d**) Microarchitecture of spheroids visualized by deep-tissue two photon excitation (2PE) microscopy. EpCAM was detected by specific fluorescent antibodies (red), nuclei were stained with Hoechst dye (blue). Magnification, 20-fold. (**e**) Microscopy demonstrates that Pica_WT04_ (WT) spheroids were killed and disassemble under cisplatin treatment compared to Pica_KO36_ (KO) spheroids. Magnification, 5-fold.

In order to more closely approach the tumor situation in vivo, a 3D spheroid model and viability protocol was established. Cisplatin resistance was confirmed not only in 2D conventional cell cultures but also in 3D tumor spheroids, mimicking more closely the tumor microarchitecture in patients. Here, automated high content microscopy allowed to objectively confirm the reproducible growth of tumor spheroids. Figure 4a illustrates the reliability of our method (*n* = 8) and showed representative spheroids over the cultivation period (Figure 4b). Notably, two-photon 3D microscopy was used to visualize the spheroids’ microarchitecture and confirm expression of the epithelial surface marker EpCAM (Figure 4d, Supplementary Video S1). The resistance of the VRAC-deficient cells against cisplatin as well as carboplatin is shown in Figure 3f and Figure 4c. Interestingly, VRAC-deficient, Pica_KO36_, spheroids stayed intact even after prolonged treatment with high concentrations of cisplatin, while the WT spheroids disassembled under the same treatment conditions (Figure 4e). 

### 2.4. The Drug Uptake Transporter VRAC Is a Critical Determinant for Cisplatin Resistance in Naturally Occurring Cancer Cells

**Figure 5:**
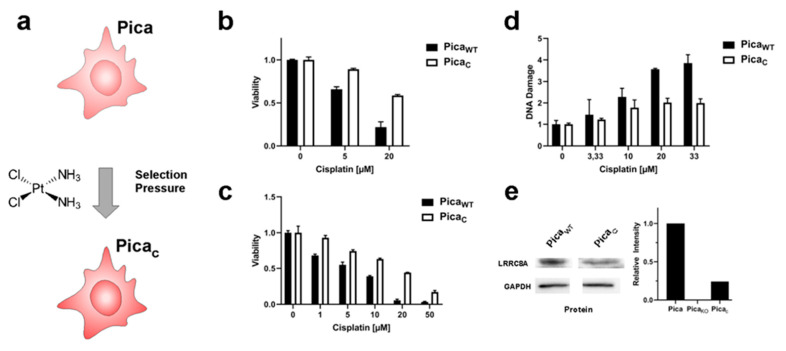
VRAC levels affect cisplatin sensitivity also in naturally occurring cisplatin-selected cells. (**a**) Scheme to illustrate the establishment of cisplatin-resistant Pica_C_ cells by chronic exposure to cisplatin. (**b**,**c**) 2D/3D Pica_C_ cell models to demonstrate cisplatin resistance. Cells (**b**) or spheroids (**c**) were drug treated (cells—48 h; spheroids—72 h), and viability was normalized to untreated controls. (**d**) Resistant Pica_C_ cells show a lower number of cisplatin-induced DNA damage events (γH_2_AX foci) per cell as automatically quantified by high-throughput microscopy. γH_2_AX foci stained by specific fluorescent antibodies. (**e**) Immunoblot analysis confers downregulation of VRAC (LRRC8A) protein levels in resistant Pica_C_ cells. GAPDH served as loading control. Data from one representative experiment of three independent experiments shown. For immunoblot analysis of Pica_KO36_ cells, refer to Figure 2b. For uncropped blots, refer to Supplementary Materials.

To independently verify our insights obtained in our engineered cell models, we additionally established a naturally occurring cisplatin-resistant Pica cell line (Pica_C_) by treatment with subtoxic concentrations of cisplatin (3–5 µM) for six months. Experiments were conducted after regular proliferation was regained to ensure a homogenous, stable cell population (Figure 5a). Notably, Pica_C_ showed similar levels of resistance compared to our VRAC KO cell line, both in 2D as well as 3D assays, also correlating with reduced numbers of DNA damage events (Figure 5b–d). VRAC expression levels were also reduced to about 25% compared to sensitive WT baseline levels, underlining again the central role and general relevance of VRAC for cisplatin chemotherapy sensitivity in HNSCC cells (Figure 5e). 

Of note, the generation of cisplatin-resistant cell culture models is not trivial as expected for an effective anticancer drug, and we did not succeed in selecting cisplatin-resistant Hunkel or Deuser HNSCC cells, as used in previous studies by us and others [33].

However, we successfully established a cisplatin-resistant cell line, Fadu_CisR_, by selecting HNSCC Fadu cells with subtoxic concentrations of cisplatin (3–5 µM) for six months (Figure S6a). Similar to the results obtained in the Pica cell models, immunoblot analysis confirmed decreased VRAC expression in the cisplatin-resistant Fadu_CisR_ cells (Figure S6b), thereby confirming our results by an additional independent cell model. 

## 3. Discussion

This study aimed at profiling cisplatin resistance mechanisms and identifying biomarkers potentially suitable as drug targets or patient stratification. The development of resistance mechanisms is clearly a complex process. We hypothesized that particularly transporters facilitating active influx or efflux of platinum drugs are the most relevant candidates, as they have a direct impact on the intracellular drug concentrations causing tumor cell death (see also Figure 1a). Several drug transporters such as CTR1, OCT1, VRAC, or MRP1 were suggested to be involved in resistance to various chemotherapeutics, although the underlying mechanisms are not fully understood [13,14,16,17,18,19,34]. In line with previous reports pioneered by the Jentsch group, we argue that our in silico and in vitro data justify our decision to focus on VRAC rather than other transporters, such as MRP1, as the most relevant factor for cisplatin resistance in HNSCC [13,14,15,20]. For one, to guide the selection of clinically most relevant candidates, we first used the TCGA HNSCC datasets to correlate drug transporter expression levels with resistances in cancer patients, focusing on patients that underwent cisplatin-based chemoradiation as a first-line therapy. While we found no significant differences in therapy response depending on the expression levels for the transporters CTR1 or OCT1, low expression of the drug import channel VRAC and increased expression of the drug export transporter MRP1 significantly correlated with residual disease (Figure 1b). However, as increased expression of the cisplatin efflux transporter MRP1 is expected to result in cancer cell resistance, and its correlation with improved clinical response to cisplatin treatment cannot be explained by the current knowledge of MRP1’s pathobiological functions. One may speculate that additional, so far unknown functions of MRP1, independent of cisplatin efflux, may ultimately contribute to improved therapy response observed in patients. In contrast, low expression of the drug import channel VRAC is expected to reduced uptake of cisplatin and thus favor cancer cell survival and tumor recurrences, as we found in patients. Second, when we analyzed resistant vs. cisplatin-sensitive cell lines, the potential cisplatin transport channel candidates CTR1, OCT1, or MRP1 were neither up- nor downregulated (Figure 2f) in contrast to the downregulation of VRAC further underlining VRAC’s relevance for drug resistance in HNSCC. However, we agree that it might be interesting for the field to further explore so far unknown functions of MRP1 contributing to improved therapy response.

We could strongly confirm in vitro the clinical observation that the response to platinum treatment is dependent on VRAC-mediated drug uptake. We generated an LRRC8A-KO cell line lacking VRAC channel expression, which is significantly less susceptible to treatment with the platinum-based drugs cisplatin and carboplatin. Notably, rescued expression of LRRC8A and thus reconstitution of VRAC channel function significantly resensitized the resistant cells to cisplatin (Figure S5). These data provide independent strong evidence that mainly VRAC function is key for mediating cisplatin sensitivity.

Collectively, the mechanistic insights from our in vitro data help to understand our clinical observation that patients with low VRAC expression in their tumors develop resistances, respond less to chemotherapy, and thus, suffer from a higher residual tumor burden when compared to patients with high VRAC expression levels (Figure S2a,b).

Our findings could further be strengthened by appropriate animal models. However, transgenic LRRC8A knockout mice show multiple physiological impairments and defects [35] and thus seem not to be appropriate models to further investigate cisplatin resistance. Hence, we established and applied our 3D cancer spheroid model, mimicking not only more closely the tumor microarchitecture in patients but also following requests to reduce animal experiments as far as possible following the 3R principles (Replace, Reduce, Refine).

VRAC is a heteromer constituted of six subunits and can be of differing composition between the subunits LRRC8A/B/C/D and E [15,20], of which subunit LRRC8A is the only constituting member, and channels formed from subunits LRRC8A and LRRC8D are mainly involved in the transport of platinum drugs [14,15]. In addition to its involvement in platinum drug transport, VRAC is known to be crucial for active volume regulation of cells, which seems to also affect cell migration, cell cycle processes, and also each cell’s highly regulated apoptosis cascade [13]. Therefore, genetic alterations affecting VRAC components may not only lead to decreased cisplatin uptake but also changes in apoptosis induction also in other tumor entities [13]. The dual role of cell volume regulation and active transport of platinum-based drugs may be important for the cancer cells to adapt to the tumor microenvironment and fine tune cell cycle, migration, and metastasis, particularly in the absence of the cisplatin drug selection pressure [13,36,37,38]. 

Analyzing the suggested molecular networks of potential interaction partners of LRRC8A, previously known to be involved in the development of cancer (Figure S7) and cisplatin resistance (Figure S8), the Ingenuity Pathway Analysis software indicates that all subunits LRRC8B-E are suggested to be associated with cancer. Interaction partners include different proteins in various cellular localizations, including nuclear proteins such as HDAC4. In addition to facilitating the deacetylation of histones and thereby regulating genetic transcription and epigenetic repression, histone deacetylase HDAC4 could be shown to promote cancer proliferation and invasion and to be associated with poor patient outcome in esophageal cancer [39,40]. LRRC8A networks of cisplatin resistance are suggested to also include NOTCH and SNAI1, which are two transcription factors with high cancer relevance. While NOTCH signaling is centrally involved in cell proliferation, differentiation, and survival [41,42], SNAI1 is involved in the induction of EMT processes, which are critical for the shedding of circulating tumor cells and metastasis [43,44]. In this context, an involvement of VRAC expression in the promotion of metastatic growth in the absence of cisplatin therapy was suggested [38,45]. 

VRAC expression is also discussed to be (in)directly involved in EMT processes [46]. When we examined marker proteins for both the epithelial as well as the mesenchymal phenotype, the KO cells tend to show slightly enhanced expression of molecular markers favoring an epithelial phenotype, such as EpCAM and reduced N-cadherin expression. Moreover, the KO cells are characterized by a reduced migratory potential (Figure S9) in wound assays and high VRAC expression levels seems associated with increased perineural sheath infiltration in the TCGA HNSCC patient cohort (*p* = 0.02). Taken together, these results signal a potential role of high VRAC expression to promote the migratory potential of HNSCC tumor cells, leading to metastasis. However, although these pathways may influence overall patient survival, it is mechanistically not resolved how these signaling cascades and their individual players do directly contribute to cisplatin resistances, which may be considered a limitation of our study and of the field. Clearly, a still unresolved limitation of bioinformatical interaction partner prediction software is the need to confirm the results by independent experimental assessment. In addition, a limitation of most large ‘omics’ studies, including ours, is the fact that corresponding non-malignant tissue, primary tumor, as well as metastases are not always available from the same patient, and treatment information often is not available. For example, in the HNSCC cohort of *The Cancer Genome Atlas* (TCGA), 565 tumors and only 50 normal adjacent tissues (i.e., 10%) were analyzed, which may preclude the identification of unknown signaling pathways and/or biomarkers. However, there is agreement in the field that besides these limitations, the high number of samples analyzed seem to outweigh these potential limitations, and the TCGA data sets formed the basis for numerous follow-up studies. We are unaware of large data sets in which corresponding non-malignant tissue, primary tumor as well as metastases have been analyzed from the same patient for all included patients, which can be considered a potential general limitation for the whole field. Notably, not only VRAC’s importance for cisplatin sensitivity seems to be masked when regarding solely overall survival profiles of very diverse patient collectives with various disease (progression) and therapy backgrounds. It is understood that these limitations apply to other previously suggested resistance proteins, such as MRP1. Only the selection of a relevant subset of data is able to reveal correlations of molecular profiles with clinical properties such as cisplatin sensitivity, as shown by our study. In addition, it would be interesting to further define the type of molecular, chemical, or physical signals (e.g., toxic agents in tobacco, HPV, DNA damage, treatments, signaling cascades, oncogenes, etc.) which cause VRAC downregulation. Concerning the relevance of the observed correlations for HNSCC, LRRC8A expression is particularly high compared to other cancer subtypes (*n* = 565, Figure S2d) and seems to also depend on the tumors’ anatomical localization (Figure S2c). Interestingly, we found that VRAC expression was significantly lower in HPV-positive (p16-positive) tumors (Figure S10a). In addition, these patients seem to respond better to chemoradiation (Figure S10b) and showed a better overall survival (Figure S10c). However, so far, there are neither data nor hypotheses explaining how the HPV status could be linked to VRAC expression and tumor pathobiology. Hence, among various potential mechanisms, a cross-talk of p16 signaling and VRAC function or the impact of the VRAC transporter during HPV infection need to be investigated in detailed follow-up studies.

**Figure 6:**
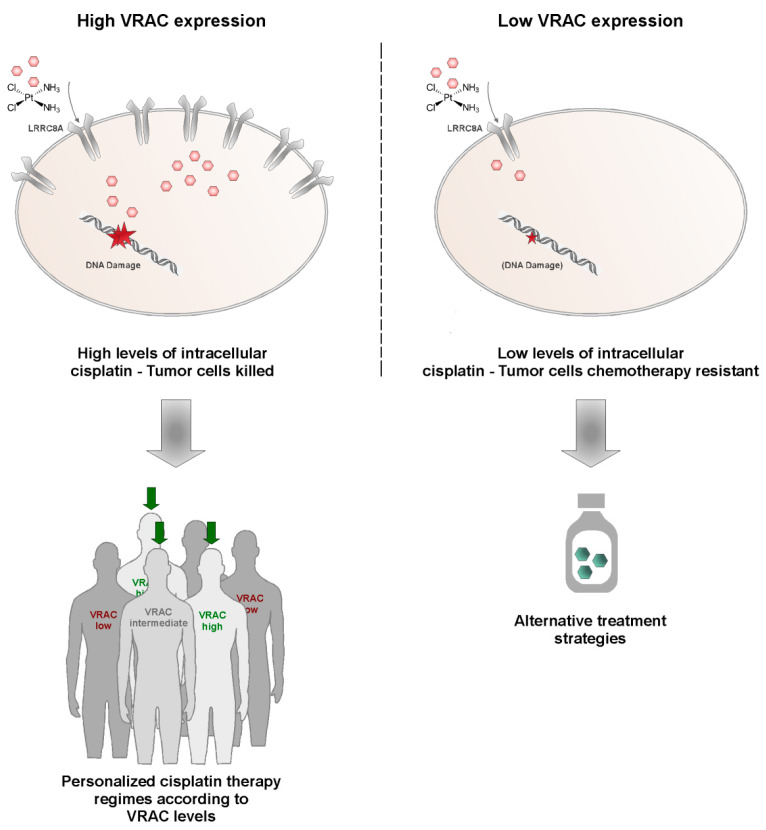
Exploiting VRAC expression levels as a strategy for personalized medicine in HNSCC. While tumors expressing high VRAC levels (green) are likely to be killed by cisplatin therapy, low VRAC levels may signal the development of resistance and thus may indicate the need for alternative treatment strategies. VRAC-based patient stratification could improve success rates in first-line chemotherapy.

Aiming at increasing patient treatment success, personalized treatment approaches are currently considered one of the most promising strategies. However, bioinformatically harnessing the full potential of powerful data sets such as TCGA is only possible by a combination of clinical hypothesis formation, educated data analysis, and in vitro confirmation of the hypothesis, as exemplified here for VRAC channel expression in HNSCC. Prospectively, personalized treatment approaches will certainly benefit from improved understanding of such personal tumor predispositions, which might signal already early the need for alternative treatment strategies (Figure 6). Such strategies might involve other drugs in clinical use, such as paclitaxel or doxorubicin, which did not show any change in effectiveness upon differential VRAC expression in our study. Alternative strategies may include nano-formulated cisplatin, which is expected to enter tumor cells independent of the VRAC transporter to cause cancer cell death. As such formulations are currently already under (pre)clinical investigation, the findings of our study may aid in selecting clinical study populations based on VRAC expression [47,48,49,50,51]. However, despite the current enthusiasm on nanomedicals, their clinical applicability and superiority compared to ‘standard’ drug formulations needs to be based on a mechanistic understanding of their advantages.

Clearly, VRAC’s pathobiological and clinical relevance concerning disease progression and therapy responses in HNSCC and other malignancies needs to be examined in detail in large prospective studies. In addition, we do not wish to postulate that VRAC is ‘the’ unique biomarker predicting (cisplatin) drug resistance for HNSCC but needs to be considered in the context of other potential biomarkers reported so far. Despite these limitations, we feel that our results strongly suggest to exploit VRAC as a potential drug target as well as a personalized prognostic biomarker to improve current and future treatments of HNSCC patients.

## 4. Materials and Methods

### 4.1. Chemicals and Reagents

Unless stated otherwise, chemicals were purchased from Sigma Aldrich/Merck (Darmstadt, Germany). Cell culture media and reagents were sourced from Gibco/Thermo Fisher Scientific (Dreieich, Germany). Disposables were purchased from Greiner Bio-One (Frickenhausen, Germany). Kits and antibodies were sourced and used as described in the further methods section und in Table S1. 

### 4.2. Clinical Data Analysis

Publicly available gene expression and survival datasets were obtained from the Cancer Genome Atlas (TCGA) filtering for patients with HNSCCs (TCGA HNSC). Thus, a total of *n* = 565 patients were included. Filtering for cases with detailed clinical information available who have received cisplatin as first-line chemotherapy, *n* = 41 cases could be included where indicated. Data were assessed via the USCS Xena server [52] and patients grouped according to indicated phenotypic or clinical characteristics. Final visualizations were performed with graphpad Prism.

### 4.3. Cell Culture

Pica cell line was established from laryngeal squamous cell carcinoma as described by Mack et al. [21]. Cells were cultured under standard cell culture conditions (37 °C, 5% CO_2_) in Dulbecco’s Modified Eagle’s Medium and subcultured every 3 d. Absence of mycoplasms was regularly checked via a Venor GeM Advance detection kit (Minverva biolabs, Berlin, Germany) according to the manufacturer’s instructions. Cell numbers were determined using Casy Cell Counter and Analyzer TT (OMNI Life Science GmbH & Co KG, Bremen, Germany).

### 4.4. Generation of Cisplatin Resistant Model 

LRRC8A-KO cells were prepared using CRISPR/Cas9 tools, as has been described [53]; however, plasmids were used for expression cassette delivery. Cells were transfected with plasmids (2 µg) using Lipofectamin 2000 according to the manufacturer’s instructions and potentially edited cells selected via Blasticidin treatment (Fisher Scientific GmbH, Schwerte, Germany, 2 µg/µL) for 9 d [53]. For primer sequences, see Supplementary Table S2. Then, single cell-derived cell lines were derived from the surviving gene pool via serial dilutions and checked for correct and homozygous knockout. Genomic DNA (gDNA) was isolated using the DNeasy Blood & Tissue kit (Qiagen, Hilden, Germany), while RNA was prepared with the help of an RNeasy Mini Kit (Qiagen) and transcribed to cDNA using a Transcriptor First Strand cDNA Synthesis Kit (Roche Diagnostics, Mannheim Germany). Primers for analytical PCRs were designed in order to span the target area of disruption for KO and PCR performed using Taq polymerase according to standard procedures. For primer sequences and expected band sized, see Supplementary Tables S2 and S3. Finally, Sanger sequencing was performed to ensure correct KO. Additionally, constantly selected cell lines were generated by treatment with doses of cisplatin corresponding to IC90 (5 µM) and then constant treatment (3 µM). First experiments were started after constant exposure for 6 months and reestablishment of regular proliferation. LRRC8A expression of cell lines was probed via Western blot analysis. Whole cell lysates were prepared in RIPA buffer and samples separated on 12% SDS gels as has been described at length [54,55,56]. Blotting onto activated PVDF membranes was achieved with a Trans-Blot Turbo (bio-rad, Feldkirchen, Germany) and blocking as well as antibody incubations (1 h/RT or 16 h/ 4 °C depending on antibody) was performed in 5% milk powder/PBS. Detection of luminescence signal of HRP-coupled secondary antibodies after addition of Clarity Western ECL Substrate was performed with the help of a ChemiDoc^TM^ (bio-rad). For primer sequences and details on plasmids as well as antibodies, see Supplementary Information. Original blots can be found at Figure S11.

### 4.5. Sequencing of DNA and RNA

Next-generation Sanger sequencing of DNA samples was commercially performed by starseq (Mainz, Germany), and sample analysis was performed with the help of benchling software. The absence of contamination of RNA samples with DNA was ensured by performing cDNA transcription steps without the addition of reverse transcriptase and then checking the amplification of the housekeeping gene actin in a PCR reaction. Then, RNA sequencing was performed as described in [53] and visualizations achieved with the help of graphpad Prism and Ingenuity Pathway Analysis (Qiagen, Hilden, Germany). For primer sequences, see Supplementary Information.

### 4.6. Probing Cell Viability 

To probe cell viability, cells were seeded in 96-well plates (5000 cells/well) and treated with indicated substances and concentrations (*n* = 3) starting 24 h after seeding. Then, 48 h after treatment, commercial assay CellTiter-Glo^®^ 2.0 (Promega, Walldorf, Germany) was performed according to manufacturer’s instructions and luminescent signals recorded using a Tecan Spark^®^ (Tecan Group Ltd., Männedorf, Switzerland). Signals were normalized to untreated control samples. For cell viability assays using spheroids, cells were seeded in 96-well round bottom, ultra-low adhesion cell culture plates (1000 cells/well, Corning GmbH, Wiesbaden, Germany) and initial spheroid formation was allowed (3 d). Then, spheroids were treated (*n* = 4) and viability was probed after 72 h using a commercial assay CellTiter Glo^®^ 3D (Promega, Walldorf, Germany) according to the manufacturer’s instructions. Luminescent signals were recorded using a Tecan Spark^®^ (Tecan Group Ltd., Männedorf, Switzerland) and normalized to untreated controls. 

### 4.7. Fluorescence Microscopy

Fluorescence images were acquired on an Axiovert 200 M fluorescence microscope (Zeiss, Oberkochen, Germany) or automated high content screening microscope Array Scan VTI (Fisher Scientific, Schwerte, Germany). Cells were seeded in microscopy dishes (35 mm, MatTek) or clear-bottom 96-well plates (Greiner Bio-One GmbH, Frickenhausen, Germany) and fixed with 4% PFA (20 min, RT). For immunofluorescence staining, they were additionally permeabilized via incubation with Triton-X 100 (0.1%, 10 min, RT). Antibodies were diluted in 10% FCS/PBS and incubated with samples for 1 h at RT. After extensive washing (PBS), fluorophore-labeled antibodies were incubated with samples for 1 h at RT. Finally, nuclei were stained by the addition of Hoechst 33342 (50 ng/mL in PBS) for 30 min at RT. For automated high content screening, regions of interests were created using the nucleus signal, and each sample was acquired in triplicates, imaging at least 5000 events per sample. For two photon-excitation (2PE) microscopy of spheroids, spheroids were cultured for 3 d and then fixed via incubation in 4% PFA at RT (20 min). Permeabilization was performed in PBSTD (PBS, 0.3% Triton-X 100, 1% DMSO, 1% BSA) and spheroids then incubated with primary antibodies diluted in PBSTD overnight (4 °C). Secondary antibody incubation was performed for 3 h at RT. Finally, nuclei were stained via incubation with Hoechst 33342 (50 ng/mL) for 15 in at RT. Spheroid collection and washing in between incubation steps was achieved via gentle centrifugation (100 g, 3 min). Images were acquired on a Leica TCS SP8 DIVE System (Leica Microsystems, Weimar, Germany). For details on antibodies and dilutions, see Supplementary Information.

### 4.8. Cell Migration Assay

To measure cell migration, cells were seeded into the two wells of ibidi two-well dishes (70 µL, 5 × 105 cells each, Ibidi GmbH, Gräfelfing, Germany) and incubated for 24 h until cells reached confluency. Then, the silicon barrier was removed with the help of sterile forceps, and the medium including any detached cells was changed. Consistent areas were documented periodically over 48 h via microscopy.

### 4.9. Transient LRRC8A Expression

In order to reconstitute LRRC8A expression in a KO cell line Pica_KO36_, cells were transfected with plasmid pBix-Ep containing the coding sequence of human LRRC8A or LRRC8A fused to a C-terminal FLAG tag, a constitutive EF1alpha promoter, and the puromycin antibiotic resistance gene. Plasmid and Lipofectamine 3000 (Fisher Scientific, Schwerte, Germany) were mixed according to the manufacturer’s instructions and added to the cells, which were cultured in OptiMEM medium as described [33]. To mark LRRC8A-expressing cells, plasmid pC3 coding for GFP expression was co-transfected. To exclude artefacts, a control transfection of empty plasmid pC-DNA3 and the GFP-coding plasmid was conducted in parallel. Medium was changed 4 h post-transfections to normal cell culture medium and cells were treated with cisplatin for 48 h starting 24 h post-transfection. Cells were fixed with PFA, and the number of green cells was quantified. FLAG-tag was stained by specific antibodies, and cells were analyzed both by conventional as well as by automated high content microscopy as described [57]. To determine changes in viability, CellTiter-Glo^®^ assay was performed as described.

## 5. Conclusions

Treatment success of head and neck cancers (HNC) is often hindered by tumor relapses due to therapy resistances. This study aimed at profiling potential molecular players critically involved in cisplatin resistance. Following a tiered pipeline from clinical hypothesis formation over educated bioinformatic analysis to in vitro hypothesis confirmation, we conclude that the volume-regulated anion channel VRAC is critical for cisplatin resistance in head and neck squamous cell carcinoma tumor cells and cancer patients. Our conclusion is based on the following findings: For one, low expression of the drug import channel VRAC correlates with reduced response to cisplatin-based chemoradiation and tumor recurrences in the TCGA HNSCC patient cohort. Second, VRAC’s key relevance for cisplatin resistance and specificity was confirmed by CRISPR/Cas9 knockout cells, which could be rescued by VRAC expression. Third, low VRAC expression correlated with cisplatin resistance in independent, naturally occurring, cisplatin-resistant HNSCC cancer cell models. Collectively, we suggest that VRAC should be considered a promising biomarker for cisplatin resistance in HNSCC and form the basis for prospective clinical trials to investigate its predictive power. 

## Data Availability

The cell line raw data required to reproduce these findings is available upon request.The clinical results shown here are based upon data generated by the TCGA Research Network: https://www.cancer.gov/tcga (accessed on 19 August 2021).

## Supplementary Materials

The following are available online at https://www.mdpi.com/article/10.3390/cancers13194831/s1, Figure S1: Overall survival of HNSCC patients correlating with potential cisplatin resistance candidates, Figure S2: Significance of VRAC’s expression for clinical data depends on case stratification, Figure S3: Genetic Analysis of LRRC8A-Knockout clone Pica_KO36_, Figure S4: Next-generation sequencing confirms LRRC8A-knockout in clone Pica_KO_, Figure S5: Ectopic expression of LRRC8A reconstituted VRAC channel function and significantly resensitized the resistant LRRC8A-knockout cell line Pica_KO36_ to cisplatin mediated cell death, Figure S6: Permanent cisplatin selection leads to reduced levels of LRRC8A expression in Fadu cells, Figure S7: Molecular network of LRRC8A interaction partners involved in cancer diseases, Figure S8: Molecular network of LRRC8A interaction partners involved in cisplatin resistance, Figure S9: VRAC expression impacts cellular phenotype and migratory potential, Figure S10: Correlation of LRRC8A expression and clinical characteristics depending on the HNSCC patients’ HPV status, Figure S11: Original blots. Table S1: Antibodies, Table S2: Primer Sequences, Table S3: Expected Band Sizes Genomic Analysis, Table S4: RNA Sequencing Results, Video S1: Cancer Cell Spheroid.
