# Supplementary material for: Profiling Cisplatin Resistance in Head and Neck Cancer: A Critical Role of the VRAC Ion Channel for Chemoresistance
Source: Cancers (Basel). 2021 Sep 27;13(19):4831. doi: 10.3390/cancers13194831 (PMC8508519; doi:10.3390/cancers13194831)
Supplement: Supplementary file 1 [file cancers-13-04831-s001.zip › SI_Siemer_MDPI_revised.pdf]

cancers

Supplementary Information

Profiling cisplatin resistance in head and neck cancer: a critical role of the VRAC ion channel for chemoresistance

Svenja Siemer[1], Torsten Fauth[2], Paul Scholz[2], Yara Al-Zamel[1], Aya Khamis[1], Désirée Gül[1], Laura Freudelsperger[1], Barbara Wollenberg[3], Sven Becker[1,4], Roland H. Stauber[1,*], and Jan Hagemann[1]

[1] Department of Otorhinolaryngology Head and Neck Surgery; Molecular and Cellular Oncology; University Medical Center, Mainz, Germany; svenja.siemer@uni-mainz.de (S.S); yalzamel@students.uni-mainz.de (Y.A.); ayakhamis@uni-mainz.de (A.K.); desiree.guel@unimedizin-mainz.de (D.G.); laura.freudelsperger@unimedizin-mainz.de (L.F.); rstauber@uni-mainz.de (R.S.); jan.hagemann@unimedizin-mainz.de (J.H.)

[2] BRAIN AG, Zwingenberg, Germany; ps@brain-biotech.de (P.S.); tf@brain-biotech.de (T.F.)

[3] Department of Otorhinolaryngology Head and Neck Surgery, University Hospital Klinikum rechts der Isar, Munich, Germany; Barbara.wollenberg@tum.de (B.W.)

[4] Department of Otorhinolaryngology, University Medical Center Tuebingen, Tuebingen, Germany; sven.becker@med.uni-tuebingen.de (S.B.)

*Correspondence: rstauber@uni-mainz.de, Tel.: +49-0-6131177002

Received: date; Accepted: date; Published: date

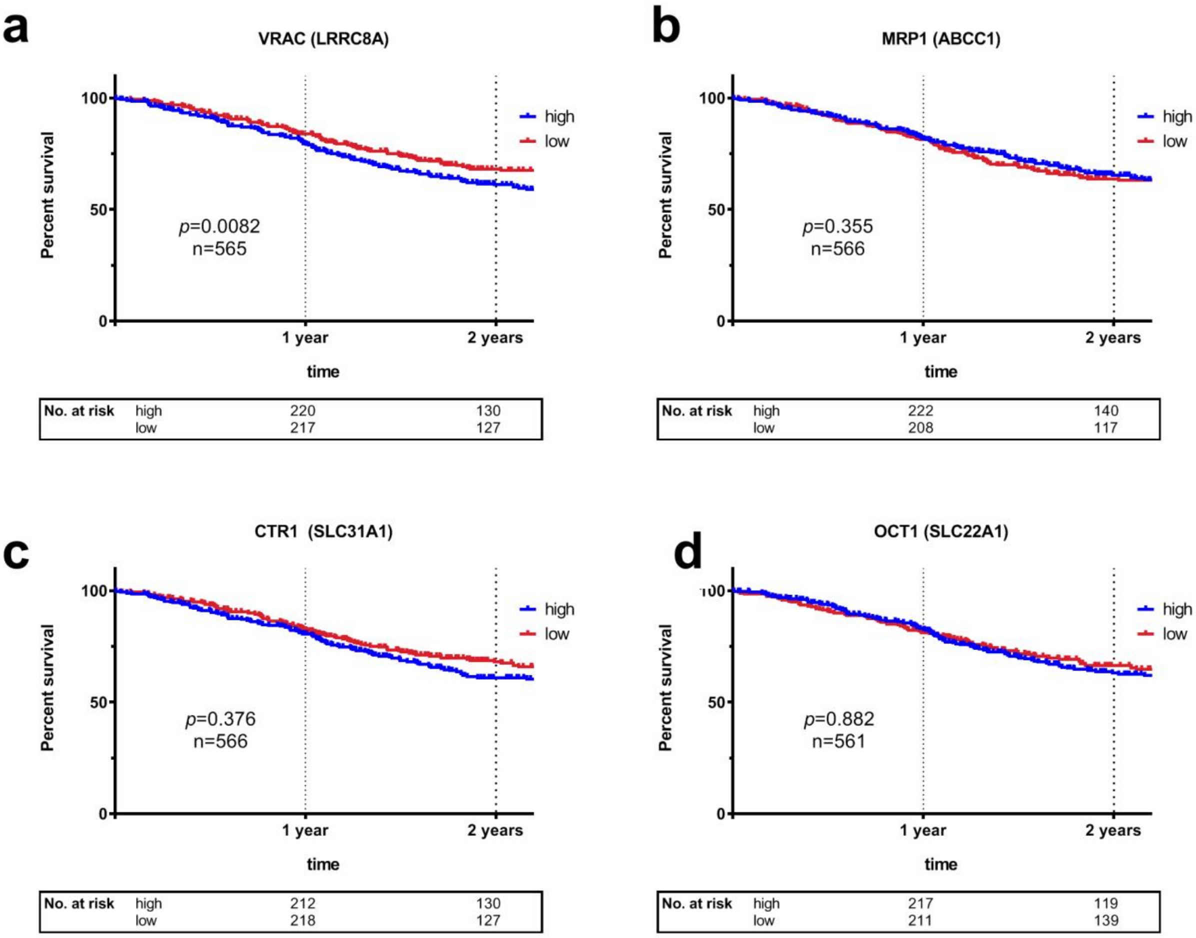

Fig. S1: Overall survival of HNSCC patients correlating with potential cisplatin resistance candidates. Overall survival of HNSCC patients depending on **(a)** VRAC (LRRC8A), **(b)** MRP1 (ABCC1), **(c)** CTR1 (SLC31A1), and **(d)** OCT1 (SLC22A1) expression levels shown by Kaplan Meier plots.

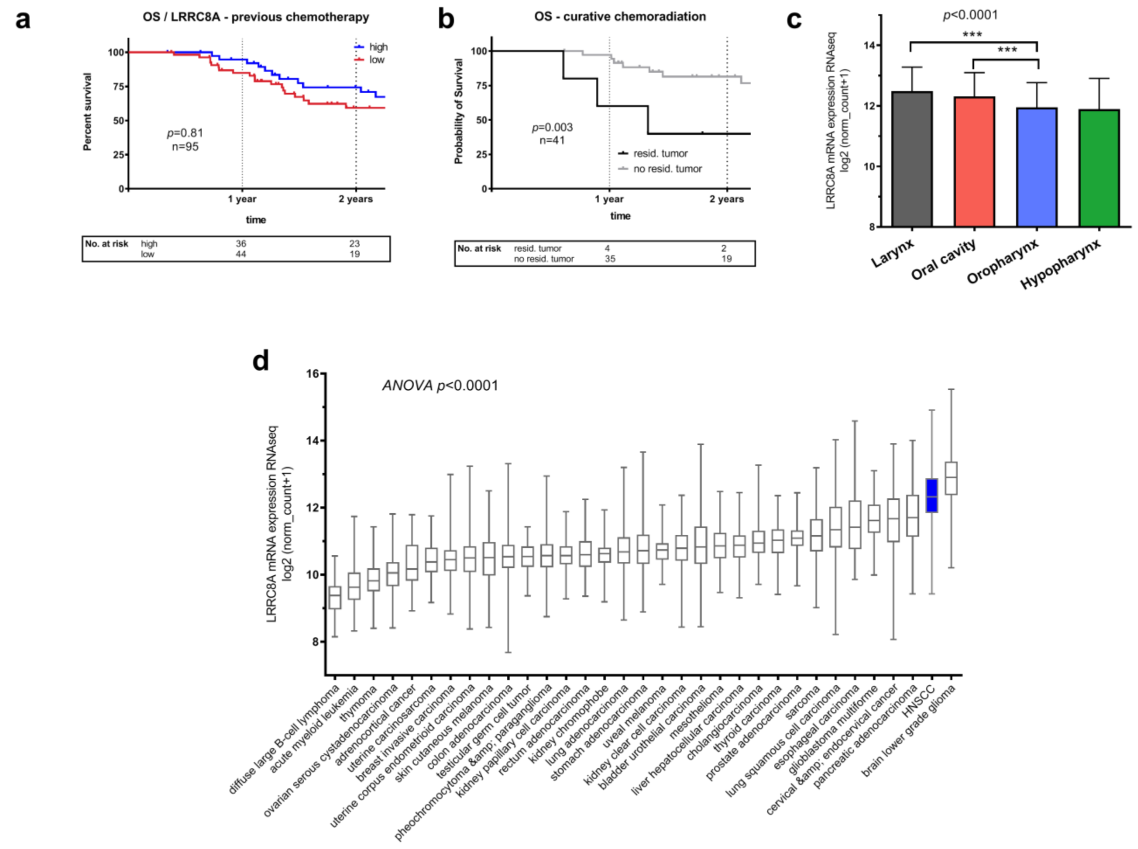

Fig. S2: Significance of VRAC's expression for clinical data depends on case stratification. (a) Overall survival of HNSCC patients, which received previous chemotherapy, depending on LRRC8A expression levels. **(b)** Overall survival of HNSCC patients, which showed or did not show residual tumors after curative chemoradiation. **(c)** Expression levels of LRRC8A across different types of cancers. HNSCC cancers show comparatively high levels of LRRC8A and are marked. **(d)** LRRC8A levels of HNSCC cancers are significantly different depending on localization.

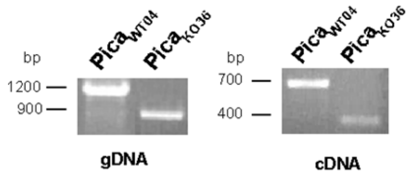

Fig. S3: Genetic Analysis of LRRC8A-Knockout clone Pica$_{KO36}$. Knockout was confirmed on gDNA and cDNA level. For primers and expected band sizes, see Supplementary Table S2 and S3.

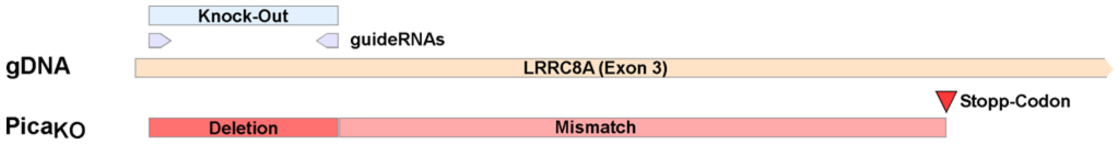

Fig. S4: Next-generation sequencing confirms LRRC8A-knockout in clone Pica$_{KO}$: Visualization of the gDNA sequencing result of the knockout clone Pica$_{KO}$. For primer sequences refer to Supplementary Table S2.

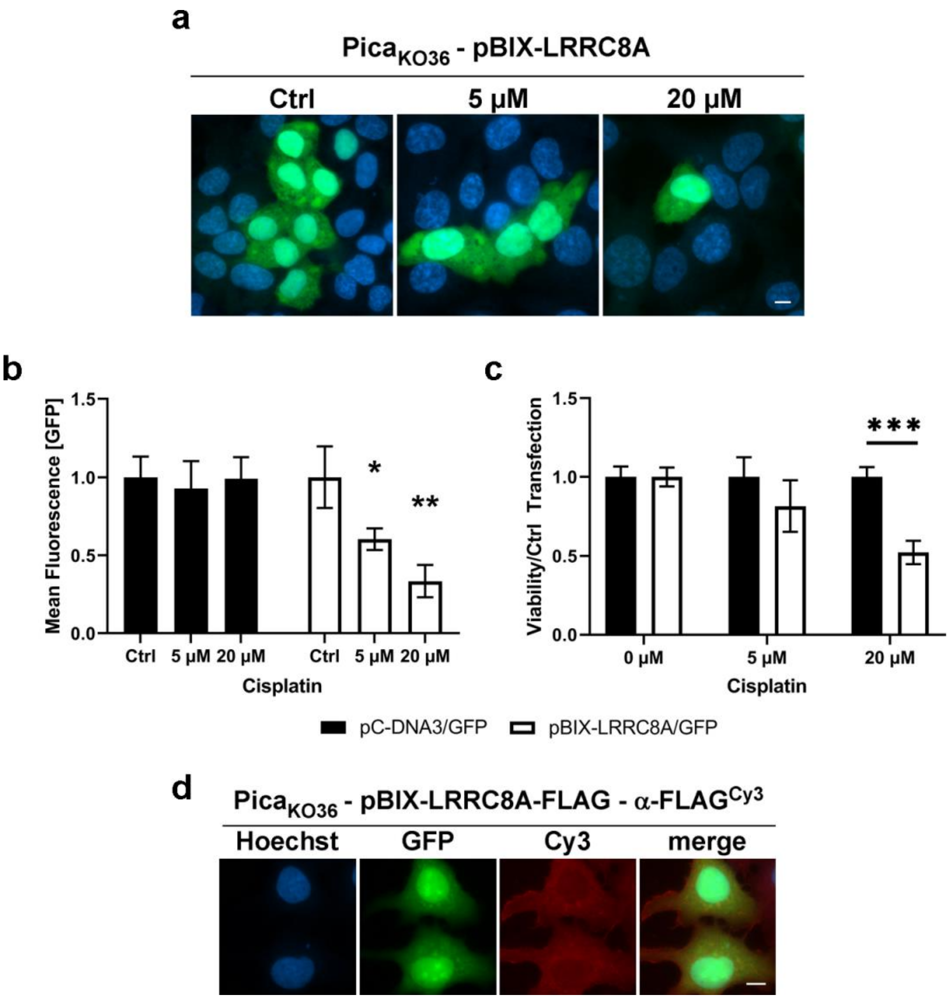

Fig. S5: Ectopic expression of LRRC8A reconstituted VRAC channel function and significantly resensitized the resistant LRRC8A-knockout cell line Pica$_{KO36}$ to cisplatin mediated cell death. (a-b) Pica$_{KO36}$ cells were co-transfected with pBIX-LRRC8A (1.8 µg) together with pC3-GFP (0.2 µg) plasmids or as control pC3-DNA3 (1.8 µg) together with pC3-GFP (0.2 µg) plasmids. 24 h later, cells were treated with indicated cisplatin concentrations for 48 h and the number of green cells quantified. **(a)** Microscopy images demonstrating that ectopic expression of LRRC8A resensitized Pica$_{KO36}$ cells to cisplatin, leading to cell death and decrease in the number of transfected green cells. **(b)** Automatic high-content microscopy to quantify the number of LRRC8A/GFP expressing cells. In contrast to the control (pC-DNA3/GFP; left), LRRC8A/GFP expressing cells (right) were killed by cisplatin concentration-dependently. **(c)** Ectopic expression of LRRC8A reconstituted VRAC channel function and resensitized the resistant LRRC8A-knockout Pica$_{KO36}$ cells to cisplatin mediated cell death. Viability was assessed by CellTiter-Glo® assay and normalized to untreated controls. **(d)** Pica$_{KO36}$ cells were transfected with pBIX-LRRC8A-FLAG (1.8 µg) together with pC3-GFP (0.2 µg) plasmids. Expression of the FLAG-tagged LRRC8A protein at the plasma membrane/cytoplasm was confirmed by anti-FLAG-staining (red). FLAG was stained with an anti-FLAG antibody and nuclei visualized by Hoechst 33342 staining. Scale bars, 10 µm. Statistical analyses by unpaired Student's t-test. *, $p<0.05$; **, $p<0.01$; ***, $p<0.005$.

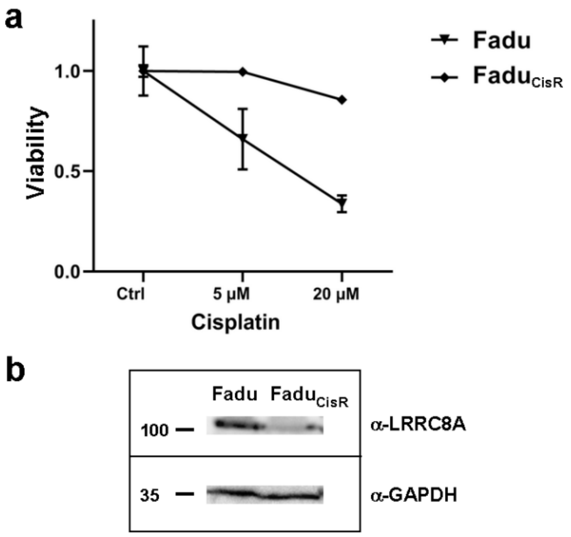

Fig. S6: Permanent cisplatin selection leads to reduced levels of LRRC8A expression in Fadu cells. (a) $Fadu_{CisR}$ are cisplatin-resistant. Cells treated for 48h and viability normalized to untreated controls. **(b)** Immunoblot analysis reveals decreased protein levels of LRRC8A in $Fadu_{CisR}$ cells. GAPDH served as loading control. MW (kD) is indicated.

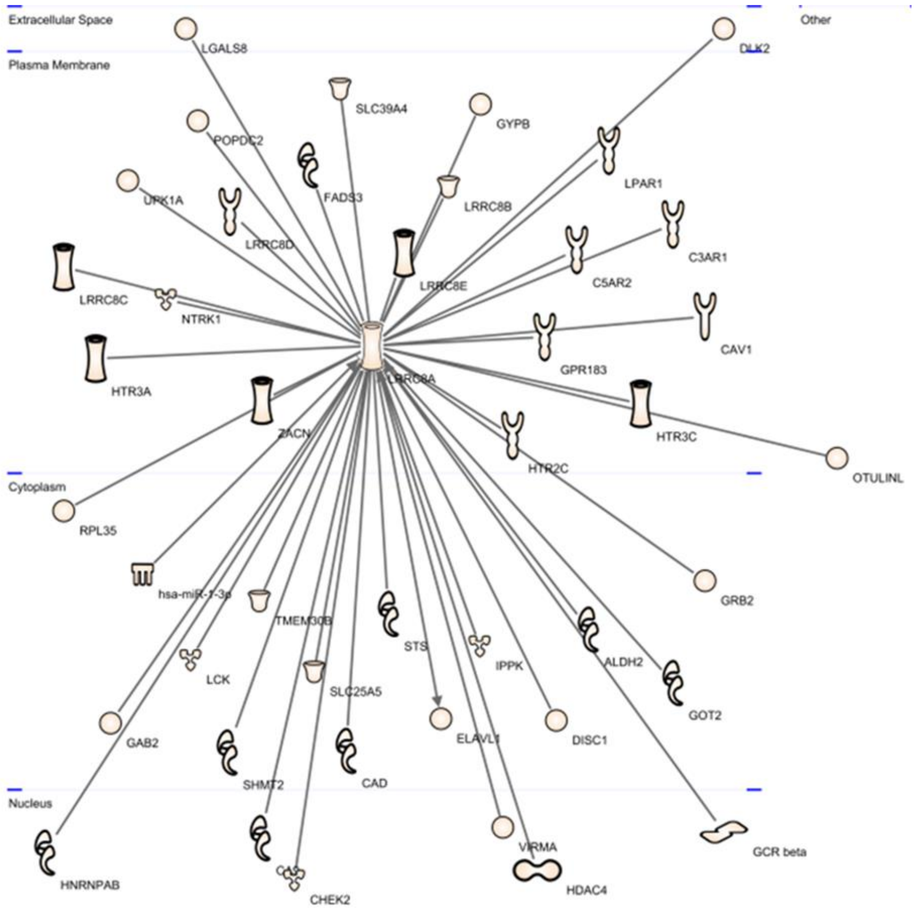

Fig. S7: Molecular network of LRRC8A interaction partners involved in cancer diseases. Ingenuity Pathway Analysis of known direct LRRC8A-interaction partners which are involved in development of cancer diseases. Cellular localization indicated.

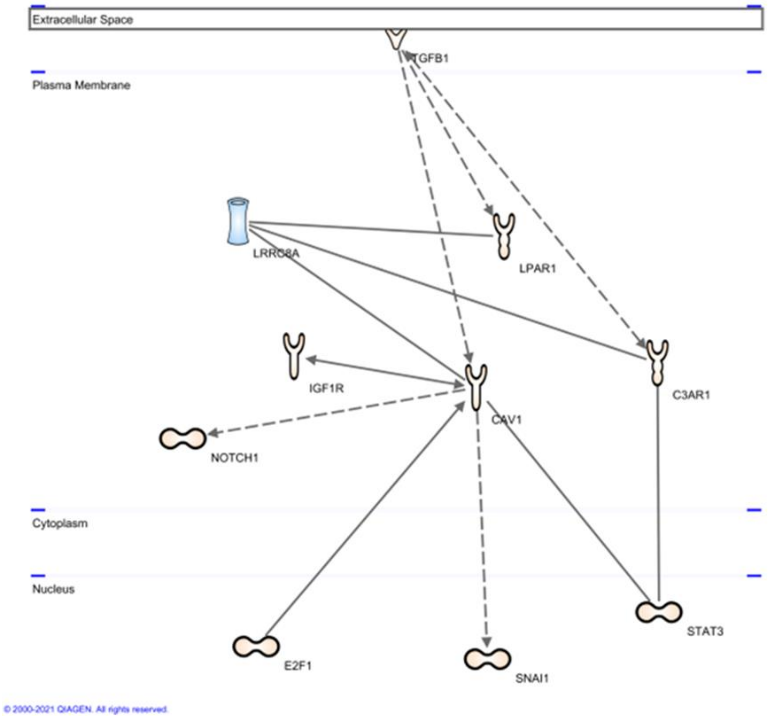

Fig. S8: **Molecular network of LRRC8A interaction partners involved in cisplatin resistance**. Ingenuity Pathway Analysis of known direct LRRC8A-interaction partners which are involved in development of cisplatin resistance. Cellular localization indicated.

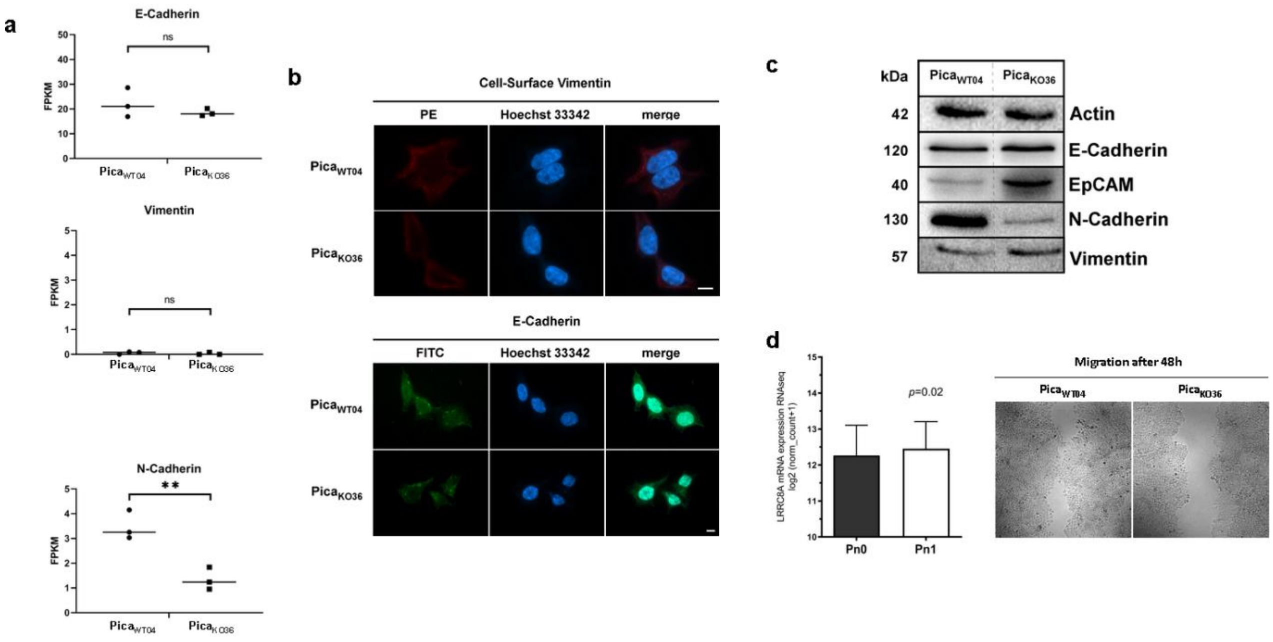

Fig. S9: VRAC expression impacts cellular phenotype and migratory potential. (a) Expression levels of EMT marker proteins in WT *versus* the VRAC-deficient KO cell line, Pica$_{KO36}$. RNA intensities as FPKM values are displayed. **(b)** Fluorescence microscopy of cell-surface vimentin (red) and E-cadherin (green) (stained with specific antibodies), nuclei stained with Hoechst (blue). Scale bar, 10 μm. **(c)** Western blot to demonstrate expression levels of indicated marker proteins. Actin served as loading control. **(d)** (Left) High VRAC expression levels may be associated with increased perineural sheath infiltration in the TCGA HNSCC patient (n=565) cohort (p=0.02). (Right) Low VRAC expression reduces the migratory potential of Pica$_{KO36}$ cells compared to Pica$_{WT04}$ cells. Cells were seeded into ibidi two-well dishes and microscopy images taken 48 h after removal of the silicone barrier.

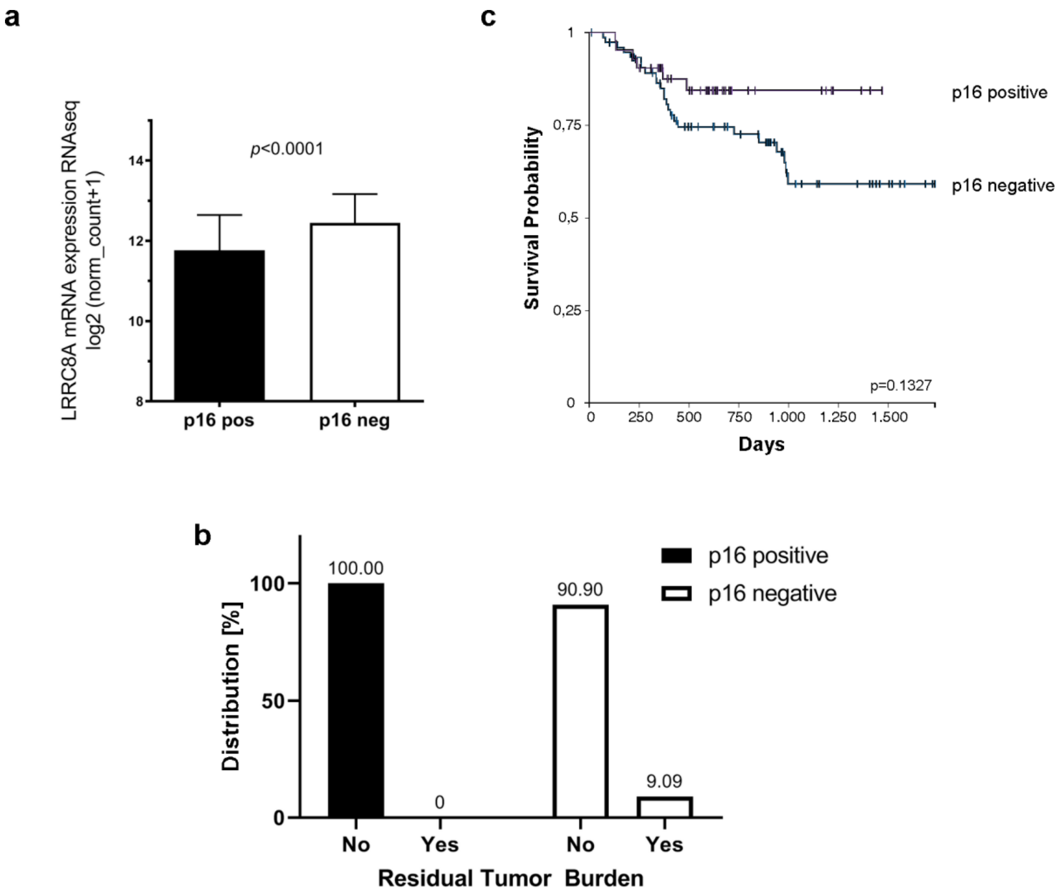

Fig. S10: Correlation of LRRC8A expression and clinical characteristics depending on the HNSCC patients' HPV status. Bioinformatic analyses were performed in the TCGA HNSCC cohort. **(a)** LRRC8A expression is significantly lower in HPV-positive (p16-positive) tumors. Bioinformatic analyses of HPV-positive *versus* -negative tumors (p16-testing as a surrogate marker for HPV-association) and LRRC8A expression were performed. **(b)** Overall survival of HNSCC patients depending on HPV status. HPV-positive patients show a better overall survival. **(c)** HPV-positive patients seem to response better to chemoradiation. Patients with HPV-negative tumors have a higher likelihood of recurring tumor disease after treatment.

Supplemental Tables

Tab. S1: Antibodies.

Antigen	Host	Manufacturer and Article Number	Dilution (x-fold)
**<u>Western Blot Analysis</u>**			
**Actin**	rabbit	Sigma Aldrich, A2066	5000
**E-Cadherin**	mouse	BD Bioscience, 610182	5000
**EpCAM/ CD326**	mouse	eBioscience; 13-9326	1000
**GAPDH**	mouse	Santa Cruz, sc-47724	3000
**LRRC8A**	rabbit	Novusbio; NBP2-32082	500
**N-Cadherin**	mouse	BD Bioscience, 610921	5000
**Vimentin**	mouse	Abnova, H00007431-M08	1000
**Rabbit (HRP coupled)**	goat	Cell Signaling; 7074	5000
**Mouse (HRP coupled)**	mouse	Cell Signaling; 7076	5000
**<u>Immunofluorescence</u>**			
**Cell-Surface Vimentin-PE**	mouse	Abnova, H00007431-MP08	200
**E-cadherin**	mouse	BD Bioscience, 610182	200
**EpCAM/CD326**	mouse	eBioscience; 13-9326	200
**FLAG**	mouse	Sigma, F3165	300
**γH2AX**	rabbit	Bethyl; A300-081A	2000
**Rabbit (Cy3 coupled)**	goat	Dianova; 111-165-003	300
**Mouse (Cy3 coupled)**	goat	Dianova; 115-165-062	300

Tab. S2: Primer Sequences

Name	Sequence (5′ → 3′)	Application
**LRRC8A_fw**	CTGGGATTACAGACGTGAAC	Characterization knockout clone
**LRRC8A_rev**	TGCTCGATCCGTGACTTG	Characterization knockout clone
**LRRC8A-cDNA_fw**	ATCCTTGGGGTTGAACCATGAT	Characterization knockout clone
**sgRNA-LRRC8A_fw**	GCTGCGTGTCCGCAAAGTAG	Guide sequence CRISPR/Cas9 LRRC8A knockout forward
**sgRNA-LRRC8A_rev**	CCGGCACCAGTACAACTACG	Guide sequence CRISPR/Cas9 LRRC8A knockout reverse

Tab. S3: Expected Band Sizes Genomic Analysis

Primer 1	Primer 2	Type of Analysis	Expected band size
LRRC8A_fw	LRRC8A_rev	PCR on gDNA	**WT: 1238 bp**
			**KO: 942 bp**
LRRC8A-cDNA_fw	LRRC8A_rev	PCR on cDNA	**WT: 679 bp**
			**KO: 401 bp**

Tab. S4: RNA Sequencing Results. Only significantly differentially transcribed genes included. All values in FPKM.

Sample/ Gene	$Pica_{WT04}$ 1	$Pica_{WT04}$ 2	$Pica_{WT04}$ 3	$Pica_{KO36}$ 1	$Pica_{KO36}$ 2	$Pica_{KO36}$ 3
HES4	29.67	21.93	16.09	18.35	8.06	10.49
ISG15	105.64	122.38	47.75	31.34	48.54	43.34
B3GALT6	14.05	13.78	14.26	13.86	9.25	8.90
MFAP2	9.06	7.11	5.10	1.14	1.13	1.61
CAMK2N1	6.20	3.33	3.05	6.99	6.78	8.55
HSPG2	6.40	1.19	1.04	2.61	0.66	0.44
MARCKSL1	47.98	43.49	39.08	25.97	20.44	23.27
NCDN	16.13	11.13	11.62	13.85	7.08	8.17
ZC3H12A	8.92	2.05	2.31	2.03	1.37	0.99
SLC6A9	19.36	9.16	10.27	8.19	7.38	5.35
PLK3	5.60	2.79	2.62	2.46	1.27	2.02
ZSWIM5	1.16	1.02	1.03	2.32	1.75	2.10
TSPAN1	62.20	69.79	66.52	95.10	134.61	108.92
CDKN2C	6.89	6.45	7.46	14.15	11.82	11.65
GADD45A	44.31	30.52	31.23	12.87	18.01	15.49
IFI44	5.09	10.45	2.63	0.50	1.49	1.32
ADGRL2	2.38	1.92	1.32	0.75	0.80	0.74
DDAH1	5.69	6.41	7.27	8.61	10.72	8.56
CNN3	24.76	30.79	25.42	12.31	20.99	18.46
F3	42.61	29.60	23.65	8.45	14.01	11.40
SORT1	7.33	9.82	10.40	6.47	6.00	4.93
PBXIP1	5.78	4.72	3.77	9.23	5.75	8.80
KIRREL	7.89	4.08	2.60	4.71	2.31	2.40
PVRL4	3.82	3.90	2.56	1.99	1.54	2.03
CEP350	2.83	3.16	2.35	4.37	3.30	3.39
GLUL	30.36	27.45	27.54	52.96	54.92	40.56
LAMC2	27.45	15.13	11.67	8.41	9.98	8.48
FAM129A	4.11	0.51	0.35	0.16	0.73	0.24
KIF21B	3.81	1.05	1.11	1.22	0.85	0.57
CD55	28.80	40.58	38.29	14.70	27.44	18.33
ATF3	2.90	2.22	1.62	0.22	1.05	1.22
CENPF	15.25	19.93	15.73	24.19	20.19	17.45
TGFB2	1.12	1.51	0.66	2.57	1.55	1.12
TFB2M	13.07	15.84	15.89	19.68	21.50	21.59
SCCPDH	2.64	3.74	4.03	0.81	1.48	1.98
KLHL29	3.55	1.76	1.07	1.69	0.87	1.04
LBH	33.14	43.63	62.06	32.22	20.38	18.81
CDC42EP3	2.78	2.34	2.17	1.46	1.20	1.41
EPAS1	21.85	26.41	16.08	17.89	14.03	12.25
BCL11A	1.89	2.43	1.25	0.56	0.44	0.69

Sample/ Gene	PicaWT04			PicaKO36		
	1	2	3	1	2	3
PELI1	4.46	3.32	2.85	6.51	5.82	6.33
TGFA	9.12	6.81	5.24	2.84	4.64	4.28
ADD2	17.85	12.67	12.31	10.91	8.89	7.23
CYP26B1	7.34	4.54	3.39	2.42	1.67	1.72
SLC4A5	3.12	4.04	4.74	1.82	2.70	2.54
MTHFD2	248.90	253.55	291.77	144.23	206.93	168.14
HK2	15.38	11.52	13.45	4.43	4.64	3.93
CAPG	19.23	18.13	17.14	7.41	12.02	13.40
CHST10	2.64	4.14	4.23	1.30	1.33	1.70
MALL	5.25	4.24	7.47	2.06	3.31	3.32
SLC20A1	17.82	18.42	13.67	10.78	11.61	9.97
IL1B	19.31	3.61	3.09	1.03	1.45	1.43
IL1A	9.58	1.09	0.57	0.30	0.52	0.62
STEAP3	11.38	9.46	10.29	8.08	7.42	5.42
ARHGEF4	6.11	4.39	4.91	4.49	2.84	2.43
FMNL2	5.76	7.10	4.66	3.13	3.96	3.04
SERPINE2	6.47	5.20	3.11	0.58	1.52	0.65
SLC4A7	4.83	4.64	3.96	2.94	2.87	1.49
SEMA3B	6.24	4.70	4.88	2.72	3.40	2.96
IFRD2	83.96	65.23	72.86	62.07	58.14	45.28
FSTL1	0.94	1.55	1.22	2.42	2.15	3.10
GOLGB1	4.53	5.66	4.39	8.16	5.99	5.60
AMOTL2	21.83	13.68	13.53	33.09	19.97	22.75
PCOLCE2	4.86	9.50	8.11	3.35	4.88	3.52
TM4SF1	23.03	30.37	20.34	8.06	11.41	8.11
GPR87	16.03	21.34	22.06	24.11	28.29	36.47
LAMP3	2.46	1.67	1.20	0.59	0.60	0.41
SOX2	9.39	7.02	5.67	1.19	0.31	0.57
ST6GAL1	1.70	1.61	1.07	0.42	0.63	0.37
CLDN1	19.57	10.60	6.60	6.96	7.49	5.92
CPLX1	2.67	2.31	2.53	15.33	6.71	5.78
FGFR3	157.17	96.55	89.51	398.23	164.80	192.87
MXD4	7.82	6.07	5.94	14.43	7.92	10.37
SEL1L3	7.77	5.24	4.33	2.20	3.08	2.31
CXCL8	59.91	29.51	35.02	3.14	5.78	4.55
CXCL1	29.21	4.44	4.05	0.92	1.21	1.42
CCNG2	1.81	2.62	1.54	3.32	3.28	4.42
CENPE	2.16	5.04	4.14	4.67	4.82	4.70
TET2	1.52	1.49	1.31	2.37	1.82	1.95
LEF1	1.90	1.88	1.35	0.58	0.90	0.85
PRSS12	1.86	1.96	1.74	0.66	1.02	0.39
SLC7A11	10.58	8.74	10.94	6.47	5.22	4.52

Sample/ Gene	Pica$_{WT04}$			Pica$_{KO36}$		
	1	2	3	1	2	3
ELF2	4.49	5.22	6.11	8.15	6.81	8.92
ARHGAP10	5.27	3.21	4.22	4.54	6.72	6.89
DDX60	2.48	2.91	0.93	0.94	1.32	1.07
LPCAT1	20.19	14.32	12.90	12.42	11.36	7.66
IRX4	15.46	14.51	10.67	9.89	6.47	6.62
MYO10	7.44	4.02	4.05	3.72	3.53	2.62
RAI14	4.83	4.63	5.01	2.60	3.23	2.57
OSMR	8.31	7.42	2.89	2.72	4.53	2.98
FST	25.89	23.51	28.36	7.82	6.63	5.77
POLR3G	7.37	6.78	7.54	3.40	5.13	4.05
PGGT1B	1.47	2.34	2.30	3.21	4.50	3.22
FBN2	8.36	6.28	4.41	2.54	2.58	1.68
HBEGF	3.47	0.97	1.18	0.56	0.44	0.75
FAT2	2.49	1.56	0.80	5.23	2.24	1.88
GABRP	2.46	0.93	0.63	2.90	2.26	3.14
DUSP1	4.06	3.56	3.82	1.37	1.33	1.90
STC2	39.26	24.86	27.83	14.73	16.88	10.89
DBN1	6.35	5.11	3.53	0.54	0.69	0.35
N4BP3	5.40	2.74	2.04	9.60	4.58	4.25
GFPT2	5.75	7.17	4.72	0.66	1.48	0.72
FOXQ1	2.51	2.66	2.40	2.17	0.96	1.33
TUBB2A	7.91	5.38	5.25	2.51	3.22	1.59
NEDD9	3.75	3.15	2.71	10.46	5.78	6.45
TRIM38	4.26	4.68	3.29	5.09	5.51	6.73
HLA-B	46.70	15.07	4.35	1.98	4.08	2.91
TAP2	39.60	21.83	20.42	19.39	19.10	10.36
CDKN1A	9.79	4.39	1.72	1.24	1.48	1.77
PEX6	6.00	4.70	3.20	10.99	6.01	6.84
VEGFA	38.05	22.48	23.10	17.58	14.24	15.91
ADGRF1	11.31	4.71	7.58	13.76	13.11	13.56
KCNQ5	2.29	2.21	1.52	1.19	1.36	0.71
ZNF292	1.65	1.72	1.42	2.26	1.88	2.25
FYN	4.09	2.67	2.11	0.85	1.63	1.29
GJA1	17.89	20.47	17.66	11.51	13.33	8.93
TNFAIP3	8.24	0.49	0.38	0.14	0.17	0.07
UTRN	1.05	0.85	0.48	2.07	1.49	1.55
SASH1	3.26	2.57	1.59	6.83	3.14	4.25
UST	2.93	1.96	1.90	0.96	1.04	1.00
AKAP12	9.88	5.70	6.05	0.62	1.14	0.73
SOD2	50.82	16.38	15.49	17.15	12.31	13.37
DFNA5	5.28	4.24	3.88	0.77	2.98	2.20
CPVL	7.01	10.04	8.28	2.15	4.79	2.38

**Sample/ Gene**	**Pica$_{WT04}$** 1	2	3	**Pica$_{KO36}$** 1	2	3
GARS	84.19	83.88	98.65	47.46	72.19	55.49
STK17A	17.21	17.44	16.55	9.04	13.75	10.90
IGFBP3	1.60	1.54	0.95	5.96	3.12	5.08
COBL	4.81	4.18	4.04	2.54	3.11	2.19
GRB10	28.14	28.94	25.20	17.91	19.99	15.31
ASL	16.04	14.12	15.93	11.53	11.55	8.29
SBDSP1	13.16	11.98	13.91	6.83	8.44	6.83
SEMA3E	1.19	1.26	1.33	2.12	2.04	2.48
SEMA3C	20.01	12.59	6.01	4.74	4.95	3.71
AKAP9	2.21	5.08	4.51	6.17	5.09	5.40
CDK6	5.10	3.69	2.89	1.83	1.94	1.49
NAMPT	20.53	21.47	20.45	10.43	16.47	12.68
IFRD1	14.80	14.14	14.21	6.01	8.49	7.03
MET	21.10	14.64	12.64	10.64	10.22	8.99
ZNF467	2.53	2.80	1.18	1.38	1.21	0.65
LRRC61	23.12	18.04	18.31	13.49	11.38	9.19
NEIL2	11.41	12.44	13.56	4.66	9.77	5.76
STC1	1.68	2.88	0.76	0.71	0.83	0.46
LOXL2	34.44	12.48	13.57	10.95	12.49	14.01
NRG1	3.41	1.54	2.24	0.22	0.38	0.29
PCMTD1	5.65	7.21	6.33	8.10	7.29	9.50
PLAG1	2.36	2.25	2.09	3.73	2.65	3.46
LRRCC1	3.67	4.78	5.33	5.08	7.65	6.82
CA2	6.30	3.26	3.13	0.65	2.14	0.50
DECR1	21.78	36.82	35.42	30.50	51.62	48.78
DPY19L4	8.20	9.83	7.73	11.51	11.63	10.56
NIPAL2	3.18	3.39	2.19	6.32	4.31	5.53
TRIB1	13.75	15.19	10.30	10.10	10.24	5.93
ST3GAL1	13.91	6.12	7.32	3.45	5.02	4.76
NDRG1	44.21	37.42	29.92	56.36	45.18	102.16
MROH6	2.11	1.75	1.24	5.24	2.62	2.72
FBXL6	7.65	7.35	7.75	4.84	4.71	4.29
BOP1	54.27	36.41	38.65	48.92	32.90	29.14
MFSD3	34.70	21.96	23.34	25.94	14.34	12.72
PLIN2	17.62	18.77	17.42	8.79	12.86	12.87
AQP3	40.20	27.44	24.67	5.36	9.76	5.16
ANXA2P2	24.13	27.76	27.29	15.17	19.75	18.10
CA9	4.82	3.81	3.45	7.09	4.11	16.26
FAM201A	1.81	1.88	2.63	5.91	3.54	4.65
SMC5	7.43	9.46	8.48	11.36	10.85	10.54
VPS13A	3.62	3.44	3.04	5.74	3.75	3.19
TLE1	6.58	4.08	3.07	2.88	2.64	2.81

Sample/ Gene	Pica$_{WT04}$			Pica$_{KO36}$		
	1	2	3	1	2	3
PSAT1	151.10	129.31	151.39	81.96	99.05	68.50
C9orf64	1.81	1.09	1.69	2.67	3.34	3.37
FGD3	1.78	1.06	0.99	3.35	2.77	3.08
CORO2A	11.18	7.03	6.10	4.61	5.20	4.86
COL27A1	5.95	3.98	3.36	4.60	2.39	1.96
CNTRL	2.83	3.42	2.23	4.46	3.92	4.20
PTGS1	4.62	3.60	1.73	3.67	5.93	6.52
LCN2	110.70	11.99	10.03	7.72	8.08	10.12
LRRC8A	29.36	19.88	21.06	17.63	12.20	15.18
NCS1	19.17	14.77	16.14	11.77	9.86	9.34
ASS1	155.64	68.36	75.94	32.34	41.29	34.96
FIBCD1	5.65	3.60	3.68	12.95	5.02	6.52
EGFL7	51.12	25.84	26.93	17.11	13.81	13.25
CLIC3	2.21	0.94	1.61	9.71	6.79	10.54
UAP1L1	3.06	4.10	3.18	3.01	1.54	1.83
ABCA2	10.33	7.15	7.29	23.06	9.26	10.21
IL15RA	8.40	4.09	4.24	2.86	2.14	2.40
FAM107B	9.17	8.35	6.70	3.32	5.54	2.93
RASGEF1A	6.06	7.23	5.34	3.30	3.87	3.13
ANXA8	8.43	9.52	7.34	5.33	7.00	6.70
ANXA8	14.49	10.45	10.12	9.90	10.16	9.25
DKK1	35.27	45.29	49.22	27.76	34.47	21.74
FUT11	1.04	0.80	0.44	3.72	1.82	1.52
SNCG	70.02	70.73	83.01	106.16	106.00	111.32
ADIRF	46.81	49.66	62.62	73.08	91.17	92.25
IFIT2	6.65	7.82	3.18	1.77	4.00	2.94
IFIT3	7.94	7.93	2.98	1.13	3.27	1.94
KIF20B	6.30	13.34	13.03	12.36	14.63	14.05
IFIT1	5.16	5.67	1.74	1.21	3.39	1.72
HHEX	8.38	11.09	9.62	6.28	4.98	5.53
ZNF518A	2.60	3.84	3.98	4.64	4.02	4.16
ANKRD2	33.87	16.60	29.29	54.55	47.18	45.59
NFKB2	29.57	14.65	11.17	11.90	9.86	8.91
COL17A1	3.45	1.39	0.77	0.48	0.39	0.76
FGFR2	7.85	5.60	5.52	14.67	9.93	11.30
TNNI2	14.34	26.79	31.36	50.47	48.68	59.77
TNNT3	4.55	14.50	13.79	16.73	16.82	29.14
PHLDA2	78.33	69.44	77.02	75.30	46.53	49.32
PRRG4	6.96	10.22	10.50	11.07	11.93	12.40
ABTB2	4.31	2.38	1.46	1.92	1.23	1.13
CD44	10.03	9.09	5.31	0.52	1.67	1.05
MAPK8IP1	3.19	4.01	2.90	2.43	1.97	1.70

Sample/ Gene	Pica$_{WT04}$ 1	2	3	Pica$_{KO36}$ 1	2	3
MDK	87.65	64.27	63.76	23.62	26.53	28.82
FAM111B	2.52	3.41	4.00	4.55	5.28	4.65
FAM111A	2.62	2.00	1.05	3.08	2.52	4.03
TMEM132A	47.44	17.75	16.39	33.05	12.75	12.85
RAB3IL1	8.46	3.96	4.03	1.92	1.55	1.45
WDR74	40.34	41.87	42.57	64.94	69.55	69.15
ATL3	1.93	2.76	2.79	0.73	1.35	1.51
DNAJC4	5.09	3.47	4.63	9.18	6.41	9.11
MAP4K2	4.45	2.89	2.25	2.77	2.12	0.92
NEAT1	51.86	45.62	32.75	26.55	24.16	21.64
SYT12	33.93	41.11	39.22	65.63	60.73	50.43
CDK2AP2	100.72	73.96	78.53	173.58	117.96	133.20
PITPNM1	52.14	30.29	25.58	91.01	42.09	43.79
ALDH3B2	3.08	2.95	3.43	8.42	6.80	6.96
CHKA	23.65	28.42	30.75	51.80	39.92	44.67
GAL	128.45	144.54	183.41	58.71	73.74	45.05
FAM181B	1.08	0.86	1.48	4.74	2.83	3.11
CTSC	41.89	39.20	34.39	18.05	25.87	19.70
CEP295	1.27	1.70	1.21	1.97	2.00	1.91
NPAT	2.33	2.51	3.23	3.88	3.57	3.15
ETS1	9.84	3.87	1.90	0.76	1.30	0.73
CCND2	41.60	33.42	29.01	72.87	61.02	66.07
EMP1	4.29	5.10	5.54	1.74	3.40	1.86
ARHGDIB	43.20	28.03	34.38	43.43	57.04	58.04
FGD4	1.81	2.15	2.02	3.34	2.64	2.39
PRICKLE1	5.72	5.59	5.21	3.54	3.87	2.66
RAPGEF3	12.63	6.29	7.52	25.24	12.78	11.67
WNT10B	7.28	5.20	5.36	1.71	2.24	1.45
DDN	6.97	6.14	6.89	6.17	3.38	3.23
IGFBP6	19.91	16.90	12.21	6.02	9.68	6.22
INHBE	10.64	4.48	5.16	1.06	1.16	0.97
AGAP2	0.56	0.45	0.44	3.98	2.21	3.00
FRS2	2.45	2.85	3.17	3.77	3.55	3.57
PHLDA1	21.03	12.33	9.75	4.03	6.90	4.01
CHST11	2.67	0.84	1.05	0.72	0.53	0.38
MTERF2	3.14	2.95	3.36	4.19	5.38	6.45
TXNRD1	173.22	129.01	176.78	101.03	100.02	87.57
FAM109A	7.45	4.28	3.85	16.00	8.58	5.63
OAS1	4.53	1.69	0.18	0.64	0.54	0.67
OASL	8.70	17.64	5.43	1.09	4.00	3.45
HCAR2	9.55	7.43	4.00	12.93	10.93	8.86
MMP17	10.12	3.89	3.19	4.55	2.13	2.67

Sample/ Gene	Pica$_{WT04}$ 1	2	3	Pica$_{KO36}$ 1	2	3
PSPC1	39.38	38.36	48.45	16.10	27.81	22.75
UBAC2	26.87	31.97	28.37	16.94	19.88	22.05
COL4A1	3.49	2.33	1.19	1.62	1.07	0.52
COL4A2	15.60	10.99	5.15	10.20	6.67	3.76
MMP14	22.91	12.27	8.86	26.18	20.59	20.90
CDH24	5.86	2.57	3.49	3.28	1.94	1.70
PCK2	29.12	19.38	20.64	12.61	15.14	8.60
IRF9	15.69	12.67	3.94	2.73	3.24	5.24
EGLN3	4.85	4.77	3.25	8.34	6.87	11.60
NFKBIA	126.77	38.23	36.22	38.34	33.72	39.35
DLGAP5	15.59	23.89	20.50	21.76	29.10	25.72
TRIP11	7.38	11.19	10.39	14.20	11.04	11.25
OTUB2	22.18	14.31	13.67	6.87	11.09	6.74
PPP4R4	5.91	3.12	2.35	1.08	1.99	1.60
IFI27	16.20	17.67	2.45	4.46	1.56	4.02
WARS	42.21	39.69	38.24	21.48	28.39	22.40
CHAC1	19.49	10.89	12.60	6.77	5.13	3.80
TPM1	25.82	21.85	18.88	33.52	34.93	29.14
STRA6	2.89	1.44	1.74	1.02	0.50	0.41
HOMER2	17.28	17.14	19.03	6.39	7.00	6.41
PDE8A	18.28	10.34	9.78	7.47	8.22	9.66
HAPLN3	7.57	4.66	2.57	3.05	2.07	2.44
ISG20	4.11	3.33	2.11	0.46	1.26	1.55
SLCO3A1	4.36	1.45	0.76	0.77	0.62	0.79
SYNM	1.85	1.98	1.73	1.15	1.05	0.63
HAGHL	12.50	9.18	8.52	8.33	4.48	5.51
KREMEN2	8.65	1.26	2.12	1.99	1.31	1.25
PKD1P1	15.04	6.79	6.59	17.58	6.18	5.63
SMG1P2	3.86	3.59	3.43	4.16	3.90	3.01
SLX1B	36.77	35.52	25.17	37.33	19.60	19.05
SMG1P2	0.00	0.91	1.88	0.00	1.08	1.64
GPT2	44.74	28.88	32.59	25.76	23.17	16.17
IRX3	8.95	4.80	4.43	5.34	3.12	3.37
LPCAT2	3.04	3.48	3.11	1.56	1.63	1.72
MT1X	29.54	60.69	36.74	14.21	13.90	24.63
MT2A	498.77	467.30	304.90	239.70	251.49	339.58
KIFC3	29.47	25.72	24.93	50.28	37.86	38.69
CDH5	10.93	7.53	7.14	22.57	15.58	9.42
ST3GAL2	4.87	3.62	2.26	2.32	2.19	1.44
ATP2C2	3.26	1.62	1.94	1.06	0.69	0.71
TUBB3	12.75	9.90	8.27	7.54	4.57	6.56
GGT6	7.74	6.08	5.58	14.94	8.11	12.20

**Sample/ Gene**	**Pica**$_{WT04}$ 1	2	3	**Pica**$_{KO36}$ 1	2	3
ACADVL	53.56	38.84	35.62	25.02	30.77	29.34
KCTD11	4.22	2.81	3.47	9.67	6.94	6.75
NEURL4	9.70	6.41	6.90	16.98	12.60	10.14
CENPV	14.51	11.20	17.95	7.83	7.89	8.37
SLC47A2	13.75	10.75	8.71	21.66	19.28	20.63
ALDH3A1	60.51	24.55	39.65	7.30	10.87	9.02
CDK5R1	2.12	2.06	2.24	1.47	0.99	0.79
SLFN5	5.47	1.95	1.09	1.65	1.11	1.20
SLFN13	3.92	4.72	3.63	6.09	5.65	5.71
SLFN11	3.14	3.55	2.59	5.37	5.98	5.74
MRM1	13.36	11.99	11.84	9.44	8.45	6.42
PGAP3	4.30	3.18	3.08	6.56	4.94	6.21
IGFBP4	12.66	15.29	10.42	4.05	3.35	1.90
TNS4	16.86	10.48	7.66	20.07	17.29	14.95
NAGLU	11.62	8.21	7.93	7.74	5.37	4.90
VAT1	16.05	11.83	10.57	6.31	7.63	6.84
HOXB9	3.00	2.25	2.13	0.61	0.69	0.42
MRC2	4.21	2.51	1.13	1.47	0.84	1.04
LINC00674	2.18	2.60	2.61	3.38	3.30	3.66
SOX9	11.56	11.10	7.68	8.82	7.81	4.06
LINC00673	14.48	12.81	11.15	6.34	9.37	6.66
TRIM47	21.16	13.55	16.44	15.22	10.33	10.12
CYGB	2.77	0.77	0.78	4.00	2.47	2.79
ST6GALNAC2	3.05	1.45	3.36	5.83	4.44	6.94
TIMP2	4.11	2.60	1.49	1.13	1.11	1.80
TBC1D16	8.92	4.12	4.80	4.98	2.65	2.07
RAC3	22.76	16.61	20.07	14.51	11.81	12.48
PYCR1	124.18	76.44	87.81	71.65	60.96	48.43
METRNL	9.59	8.66	5.57	2.72	4.16	2.57
TWSG1	6.89	7.87	9.47	11.70	11.19	11.79
ANKRD12	1.38	2.73	2.23	2.52	2.92	2.69
CDH2	3.03	4.15	3.25	1.24	1.84	0.95
FSTL3	3.61	2.31	1.52	1.02	1.02	1.12
GAMT	41.93	30.01	31.58	24.79	21.78	24.53
FSD1	7.58	5.68	4.73	2.60	1.95	1.91
C3	33.25	1.94	0.51	0.67	1.03	0.72
ZNF358	5.57	3.38	2.99	14.26	9.66	12.05
ICAM1	42.52	8.53	5.89	7.39	6.92	5.82
NFIX	6.71	5.62	4.92	3.63	2.97	2.68
PALM3	0.52	0.70	0.80	3.97	2.74	2.54
ADGRL1	8.82	4.07	4.40	4.95	2.58	3.57

Sample/ Gene	Pica$_{WT04}$			Pica$_{KO36}$		
	1	2	3	1	2	3
NOTCH3	7.56	5.74	5.32	4.38	2.55	2.90
GDF15	168.63	69.38	58.21	82.08	57.33	47.92
PDCD2L	28.57	27.41	29.90	18.59	21.06	14.58
LRFN1	5.33	2.96	3.94	2.27	1.28	1.21
BLVRB	54.29	35.57	46.40	66.26	69.38	77.89
TGFB1	4.50	3.57	1.82	0.21	0.30	0.47
RELB	9.60	3.64	2.84	2.99	1.59	3.07
IGFL1	132.78	34.46	25.10	10.80	16.56	29.66
ALDH16A1	13.96	8.15	7.27	5.27	5.18	3.41
KLK6	49.85	68.33	81.35	38.01	47.94	30.67
TRIB3	82.92	53.09	63.16	50.17	45.49	33.67
RSPO4	3.07	1.56	1.72	0.33	0.27	0.48
C20orf194	1.80	1.79	1.26	3.22	2.17	2.87
JAG1	23.55	17.09	12.60	9.73	7.64	5.19
GINS1	19.50	27.66	31.44	33.06	39.01	43.62
ZNF337	7.33	8.21	7.52	12.15	12.30	10.98
SDC4	64.39	30.84	28.53	20.91	26.38	28.44
CYP24A1	13.22	7.43	5.12	1.58	1.25	2.73
BMP7	3.21	1.92	1.73	0.50	0.33	0.36
HELZ2	9.38	3.70	1.78	4.43	1.84	1.75
ARFRP1	17.38	14.81	15.65	9.24	8.32	7.53
RGS19	8.40	5.69	6.48	6.79	2.29	4.34
C2CD2	5.69	5.09	5.12	14.08	11.38	12.42
RIPK4	15.06	11.93	13.48	28.50	19.25	21.49
ZBTB21	2.53	3.09	2.99	4.34	3.74	3.38
MX1	4.72	3.54	1.10	0.49	1.10	0.96
CBS	23.60	18.28	20.64	13.13	13.93	10.93
PDE9A	5.07	3.52	4.31	1.64	1.03	0.58
SLC19A1	39.77	26.57	27.14	33.95	19.64	14.03
ZDHHC8	4.53	2.84	1.63	1.13	0.67	0.83
MN1	1.10	1.52	0.82	3.86	2.18	2.09
LIF	43.76	25.94	34.73	21.84	17.31	25.74
HMOX1	12.11	5.77	4.95	3.45	3.16	3.71
C1QTNF6	22.73	19.58	15.98	37.97	27.44	31.72
GCAT	11.97	11.36	13.09	8.73	8.61	6.31
APOBEC3B	40.28	21.51	27.85	12.89	13.21	12.49
TBL1X	7.98	7.60	6.63	4.81	5.34	3.76
SPIN4	3.19	3.51	2.85	7.48	6.81	6.70
LINC01278	3.37	3.45	3.17	5.44	7.15	5.64
AMER1	1.34	1.17	1.43	3.06	2.51	1.95
ARHGEF9	1.34	1.42	1.20	3.41	3.34	3.26
LAS1L	17.27	20.09	22.58	31.76	31.58	28.19

Sample/ Gene	Pica$_{WT04}$			Pica$_{KO36}$		
	1	2	3	1	2	3
MSN	59.54	53.39	49.48	72.23	92.48	76.62
YIPF6	5.26	5.59	5.00	10.39	10.24	9.42
PJA1	5.05	5.65	4.14	6.67	9.40	8.38
IGBP1	12.97	14.33	17.46	26.33	33.88	37.59
PDZD11	17.95	22.81	24.53	31.86	44.84	43.11
KIF4A	8.36	10.24	8.87	16.49	18.96	17.24
FOXO4	2.96	2.55	2.09	5.99	5.87	6.68
SNX12	20.29	20.10	21.39	33.39	38.45	36.27
DLG3	8.51	9.68	9.03	15.59	17.46	17.36
MED12	4.41	4.29	3.92	8.61	7.24	5.50
ERCC6L	4.04	4.84	5.23	8.15	8.80	6.82
HDAC8	8.55	11.42	10.07	15.32	20.47	20.37
PHKA1	1.97	2.10	2.24	4.14	3.88	2.98
RLIM	4.00	4.67	4.34	8.64	8.04	7.02
ABCB7	6.26	6.90	7.12	9.26	13.81	11.92
MAGT1	11.68	13.54	13.06	19.95	23.40	20.84
RPS4X	574.02	724.51	765.61	1086.02	1522.04	1524.7
COX7B	107.45	175.30	172.03	165.89	318.18	276.70
ATRX	3.61	6.57	5.03	11.83	10.26	10.67
ATP7A	0.96	0.91	0.74	2.76	1.78	1.69
TAF9B	4.22	5.79	4.97	10.51	12.15	9.81
BRWD3	1.52	1.41	1.01	3.35	2.78	2.20
APOOL	1.99	2.00	2.25	3.93	4.60	4.37
CHM	1.41	1.96	2.01	3.35	3.68	3.48
PGK1	127.22	138.00	148.60	206.03	269.12	295.34
PCDH19	1.93	0.21	0.35	5.47	2.97	2.61
SRPX2	2.94	1.25	0.62	5.43	4.73	6.69
TSPAN6	6.78	6.11	6.39	10.40	15.96	11.34
DIAPH2	2.13	2.87	2.72	4.21	5.31	5.08
CSTF2	12.98	11.47	12.79	23.46	22.44	19.22
CENPI	1.60	1.33	1.60	2.87	3.88	2.87
TRMT2B	3.87	4.11	3.82	6.60	6.67	5.94
NONO	109.58	128.99	128.10	192.37	233.32	233.17
TCEAL8	13.19	19.20	18.46	21.75	40.42	43.04
WBP5	14.89	20.68	15.96	22.42	43.63	41.06
TCEAL1	2.48	4.11	4.45	7.20	8.08	11.69
NGFRAP1	97.12	105.60	89.54	142.49	177.40	180.96
RPL36A	273.09	435.56	482.76	444.18	858.75	971.18
TCEAL4	18.84	28.77	25.61	28.78	50.19	54.13
CXorf57	0.61	1.00	1.06	1.57	2.69	1.77
FAM199X	6.37	7.49	6.64	13.63	12.13	10.81
NUP62CL	2.17	2.28	2.11	4.27	5.84	4.44

Sample/ Gene	Pica$_{WT04}$			Pica$_{KO36}$		
	1	2	3	1	2	3
RBM41	1.39	2.87	2.38	3.52	3.39	3.01
PRPS1	20.57	22.22	23.84	33.21	44.52	36.40
PSMD10	31.24	43.55	43.87	54.00	78.43	70.89
NXT2	3.41	2.81	2.88	5.96	6.14	7.08
ACSL4	5.11	7.61	7.63	9.79	13.10	9.71
TMEM164	3.32	3.09	3.37	7.50	7.43	6.49
AMMECR1	2.89	2.95	2.95	5.60	4.63	4.52
ALG13	2.72	3.57	4.55	5.05	6.71	6.92
MORF4L2	83.41	109.89	114.89	125.46	173.62	168.46
KLHL13	3.04	4.10	3.58	7.57	8.06	7.38
IL13RA1	9.03	9.31	8.29	13.84	21.45	17.58
PLS3	35.05	40.61	36.47	47.87	68.28	56.76
UPF3B	22.61	33.22	35.74	33.88	49.19	42.22
ZBTB33	12.12	14.31	14.13	19.92	19.78	18.25
C1GALT1C1	15.94	19.02	19.67	21.21	27.74	27.79
THOC2	11.81	16.09	14.32	17.95	18.47	18.48
XIAP	12.03	13.53	11.67	17.10	16.57	16.54
ELF4	16.65	14.93	15.33	26.85	23.31	22.16
RBMX2	17.96	22.52	22.78	25.43	39.56	36.35
PHF6	13.64	19.04	19.45	27.32	28.41	24.12
STAG2	25.99	31.07	27.00	40.98	40.04	37.02
MBNL3	1.72	2.61	1.75	4.32	3.45	3.08
SLC9A6	5.15	4.85	5.18	6.94	7.71	7.92
VGLL1	1.62	0.19	0.81	5.23	4.33	6.91
FHL1	17.96	14.14	12.45	7.14	10.59	8.95
ATP11C	2.37	3.13	3.20	5.18	4.67	4.53
LDOC1	14.79	10.62	12.27	39.52	30.21	29.74
IDS	5.36	3.80	5.22	10.91	8.67	7.65
CXorf40B	10.28	9.01	12.87	16.10	18.17	17.58
TMEM185A	5.90	3.70	4.84	11.77	9.99	10.37
FMR1	5.99	7.86	7.45	12.92	14.13	13.77
CD99L2	1.42	1.54	1.82	3.30	2.78	2.75
MTMR1	10.31	11.33	9.91	21.13	20.90	22.18
VMA21	4.45	7.37	7.64	10.44	13.39	11.28
HMGB3	14.58	19.66	20.85	29.27	35.76	35.81
CETN2	13.64	27.37	26.37	29.16	43.59	46.76
ZNF275	2.04	2.09	2.03	4.00	2.98	3.23
NSDHL	13.05	19.83	22.43	33.37	35.97	42.32
MAGEA4	126.45	154.44	166.61	230.23	268.43	261.35
FAM58A	8.55	9.48	9.13	20.85	17.30	16.01
HAUS7	12.30	10.17	13.25	27.15	23.75	22.45
SLC6A8	1.97	1.94	1.59	4.62	2.90	5.77

**Sample/ Gene**	**Pica$_{WT04}$**			**Pica$_{KO36}$**		
	1	2	3	1	2	3
ABCD1	4.31	2.81	3.05	11.57	6.13	6.66
PLXNB3	1.25	0.59	0.61	3.76	1.80	1.68
L1CAM	2.18	1.55	1.63	10.53	7.21	4.87
BCAP31	131.41	146.12	159.71	258.48	301.59	281.43
NAA10	46.31	40.34	51.56	75.41	82.40	80.35
SSR4	86.44	93.87	96.67	139.35	180.95	186.47
TMEM187	4.81	4.17	4.02	9.15	8.23	10.11
MECP2	10.09	7.92	8.49	22.05	19.12	18.42
IRAK1	99.68	57.29	69.12	208.14	119.31	121.26
FUNDC2	14.81	21.49	20.50	21.23	31.42	28.54
BRCC3	9.27	10.16	9.51	16.04	16.88	16.49
CLIC2	0.48	0.46	0.49	1.03	2.18	2.00
